# Nanotechnology in combating biofilm: A smart and promising therapeutic strategy

**DOI:** 10.3389/fmicb.2022.1028086

**Published:** 2023-03-03

**Authors:** Yugal Kishore Mohanta, Ishani Chakrabartty, Awdhesh Kumar Mishra, Hitesh Chopra, Saurov Mahanta, Satya Kumar Avula, Kaustuvmani Patowary, Ramzan Ahmed, Bibhudutta Mishra, Tapan Kumar Mohanta, Muthupandian Saravanan, Nanaocha Sharma

**Affiliations:** ^1^Department of Applied Biology, School of Biological Sciences, University of Science and Technology Meghalaya (USTM), Techno City, Meghalaya, India; ^2^Indegene Pvt. Ltd., Manyata Tech Park, Bangalore, India; ^3^Department of Biotechnology, Yeungnam University, Gyeongsan, South Korea; ^4^Chitkara College of Pharmacy, Chitkara University, Rajpura, Punjab, India; ^5^National Institute of Electronics and Information Technology (NIELIT), Guwahati Centre, Guwahati, Assam, India; ^6^Natural and Medical Sciences Research Centre, University of Nizwa, Nizwa, Oman; ^7^Department of Physics, Faculty of Science, Kasetsart University, Bangkok, Thailand; ^8^Department of Gastroenterology and HNU, All India Institute of Medical Sciences, New Delhi, India; ^9^AMR and Nanotherapeutics Laboratory, Department of Pharmacology, Saveetha Dental College, Saveetha Institute of Medical and Technical Sciences (SIMATS), Chennai, India; ^10^Institute of Bioresources and Sustainable Development, Imphal, Manipur, India

**Keywords:** microbial biofilms, nanotechnology, anti-biofilm activity, medical biofilm, food biofilm

## Abstract

Since the birth of civilization, people have recognized that infectious microbes cause serious and often fatal diseases in humans. One of the most dangerous characteristics of microorganisms is their propensity to form biofilms. It is linked to the development of long-lasting infections and more severe illness. An obstacle to eliminating such intricate structures is their resistance to the drugs now utilized in clinical practice (biofilms). Finding new compounds with anti-biofilm effect is, thus, essential. Infections caused by bacterial biofilms are something that nanotechnology has lately shown promise in treating. More and more studies are being conducted to determine whether nanoparticles (NPs) are useful in the fight against bacterial infections. While there have been a small number of clinical trials, there have been several *in vitro* outcomes examining the effects of antimicrobial NPs. Nanotechnology provides secure delivery platforms for targeted treatments to combat the wide range of microbial infections caused by biofilms. The increase in pharmaceuticals’ bioactive potential is one of the many ways in which nanotechnology has been applied to drug delivery. The current research details the utilization of several nanoparticles in the targeted medication delivery strategy for managing microbial biofilms, including metal and metal oxide nanoparticles, liposomes, micro-, and nanoemulsions, solid lipid nanoparticles, and polymeric nanoparticles. Our understanding of how these nanosystems aid in the fight against biofilms has been expanded through their use.

## 1. Introduction

There is a significant correlation between the meteoric rise in the incidence of infectious diseases and the alarmingly high death and morbidity rates (Leventhal et al., 2015). Several researchers in the medical field have expressed their fascination with disease dynamics and the myriad of factors that might perpetuate and facilitate disease transmission (both acute and chronic). When in their planktonic state, microbes are responsible for acute infections; however, over time, they can develop strategies for survival and adapt to the harsh environments in which they are found. As a direct consequence, a sort of cellular community capable of intercellular communication is characterized as a “biofilm” (Fux et al., 2005; Aparna and Yadav, 2008). The term “extracellular polymeric substances” (EPS) refers to a matrix that enables microbes to adhere permanently to biological and non-biological surfaces, such as those found in microbial biofilms, which can represent a wide variety of species (such as an interspecies relationship between bacteria and fungi). EPS can be found in microbial biofilms and other places (Bjarnsholt, 2013; Burmølle et al., 2014). Proteoglycans, nucleic acids, extracellular proteins, and phospholipids are just some of the EPS matrix’s building elements formed by the bacteria that make up the biofilm. Other building blocks include phospholipids, extracellular proteins, and extracellular nucleic acids. When biofilms are formed, the biofilms themselves may contain mineral crystals and silt in addition to milk leftovers, blood components, or dirt. Biofilm may also contain blood components. We do not yet know how the different components of the EPS matrix interact with one another or how they contribute to the structural integrity of the matrix. On the other hand, it has been discovered that biofilms can thrive on the functions of EPS (Flemming and Wingender, 2010).

**Figure 1:**
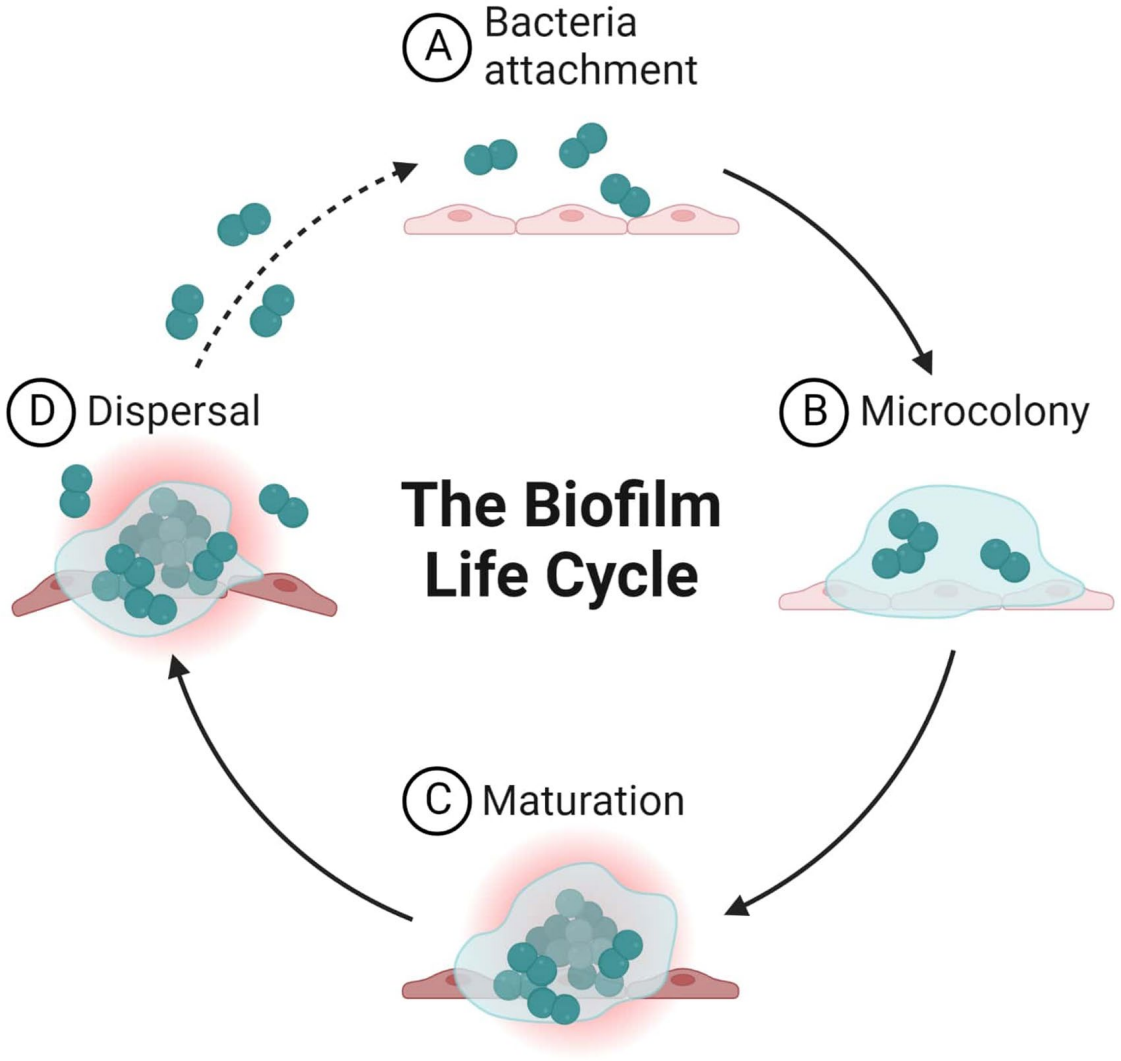
Life cycle of microbial biofilm.

The formation of biofilms (Figure 1), which typically have a thickness on the order of millimeters or even micrometers, occurs naturally around live tissues, medical gadgets, and water bodies or systems. Biofilms can range in thickness from millimeters to even micrometers. Biofilms are prevalent in the environment and on abiotic surfaces, and these biofilms, which contain additional commensal species, are referred to as biofilm multispecies (D’Acunto et al., 2015). Various organisms, including bacteria and fungi, can produce biofilm on surfaces. Specific variables involved in biofilm production, however, differ between the two types of biofilms Biofilms are in fact heterogeneous structures, with the support surface consisting of a series of discontinuous phases. This suggests the possibility of both highly-populated regions and un-colonized regions. In addition, the morphology of these cells has been found to exhibit a considerable amount of variation; different types of bacteria can have shape variations, including but not limited to filamentous, spiral, and rod morphologies as well as bacilli and cocci (Almeida and de França, 1998; Percival et al., 1998).

Effective antimicrobial therapy is frequently futile because the resistance of biofilm-associated bacteria has grown in number, making managing biofilm-related illnesses a crucial issue. This is because biofilm-associated bacteria are more resistant to antimicrobial treatment. It is currently unknown what precise mechanism leads to biofilm creation, and this will continue to be a significant focus of scientific investigation for many years to come. Despite this, it is common knowledge that the resistance and virulence of the microorganisms are connected to the makeup of the biofilm and the mechanisms at play inside it.

Due to their potential usefulness in medical treatment, nanoparticles (NPs) have recently attracted because of their ability to deliver drugs to the target site in optimal concentrations, prevent their inactivation, and boost the efficacy of their therapy with fewer adverse effects (Wang et al., 2020). The nano formulations’ small size, extensive surface area, and extremely sensitive nature allow them to penetrate biological barriers like biofilm and selectively target bacteria rather than other cells (Blanco et al., 2015; Sharma et al., 2019). The high surface area of NPs makes loading medications more effortless, and their small size allows them to penetrate biofilms and microbial cell walls (Ramasamy and Lee, 2016). Also, NPs leave the body quickly through the kidneys and stay in the blood for a long time (Blanco et al., 2015; Ramasamy and Lee, 2016).

Over the past 10 years, numerous articles have emphasized the use of nanotechnology as a medication delivery system and an alternate method of treating bacterial infections and associated biofilms. Numerous academic publications have examined nanoparticles as anti-biofilm agents, discussing their benefits, drawbacks, and changes. As an illustration, Qayyum and Khan (2016) evaluated the NPs based on their makeup and anti-biofilm activity against various bacteria. To prevent wound biofilm infections, the features, functions, and boosting factors in the antibacterial activity of NPs were extensively studied. Furthermore, the effect of EPS in nanoparticle-biofilm interactions and NPs-based drug delivery methods for regulating biofilms were investigated (Dos et al., 2018; Fulaz et al., 2019). Pircalabioru and Chifiriuc (2020) recently reviewed the development of anti-biofilm drug delivery systems based on NPs and discussed the progress in this area.

Even though NPs are becoming more effective as antimicrobials, most studies have only been conducted *in vitro*. But when clinical trial databases were searched, only very few clinical trials were found. This shows a great inconsistency between the growing research on nano anti-biofilm compounds and their use in therapy. Even though most research shows NPs to be anti-biofilm, a number of them are helpful for treatment because they might not have the properties needed for use in living organisms. Clinical applications must consider NPs’ properties, dose, biocompatibility, toxicity, and other factors to ensure that NPs work as antimicrobial and anti-biofilm with as few side effects as possible. We know that no review explicitly examines how these variables affect NPs’ clinical applications. This study aimed to look at the most recent changes in nanotechnology to treat biofilm infections and to do a detailed survey of the factors that affect the clinical and anti-biofilm effectiveness of nanoparticles (NPs). The review also highlighted the typical NPs and described how they fight off bacterial cells and biofilms as well as primary nanotechnology-based drug delivery systems as a method for managing and treating biofilms. Moreover, it talks more about the most important goals and challenges for putting this promising therapeutic strategy into practice in the real world.

## 2. Microbial biofilm: Overview

### 2.1. Bacterial biofilm

Bacterial species produce the majority of biofilms out of all microorganisms, yet some may be more capable than others when the conditions are right. Most species exhibit rapid growth, skillful adaptability, and the capability of producing compounds and extracellular structures that protect the microbes in their habitat. Because of these qualities, they can easily colonize any surface, even when things are hard (Davies, 2003). A critical factor in the growth of biofilms is the proliferation of bacteria in their planktonic phase. However, if the biofilm protects under adverse environmental conditions, its survival as a protective activity is tied to its nourishment, which relies on the continuity of its life cycle (Friedlander et al., 2013). Bacterial biofilms have some benefits, such as ecological engagement in symbiotic partnerships. There are many examples of this in nature, such as diazotrophic prokaryotic bacteria that live in plant roots and several other bacteria that live in the digestive tracts of ruminants and help break down and recycle materials that do not dissolve. Numerous complex elements play a role in the creation of bacterial biofilms. This aspect is poorly understood, which has led the scientific community to examine the complete dynamics of the intricate microbial architecture.

### 2.2. Fungal biofilm

Fungal infections are a severe concern to doctors due to their high prevalence, particularly in weakened patients. Fungal infections are common and easy to get because of host immunity, prolonged use of broad-spectrum antifungals, intravascular and urethral catheters, hemodialysis, corticosteroids, parenteral nutrition, immunosuppressive anticancer drugs, and transplants (Antas et al., 2012; Mayer et al., 2013). Fungi can live as biofilms in several niches and cause diseases. However, the site of infection is determined by exclusive parameters, including appropriate access to nutrients, the host immune response, flow conditions, pH at the entry point, and the substrate for cell adhesion and biofilm formation (Nett and R Andes, 2015).

Most of the *Candida* spp. create biofilms at different flow rates of the body fluids, which might be low (salivary flow: prosthetic stomatitis), intermittent (urinary catheters and vascular circulation), and rapid (bloodstream: fungal endocarditis; Douglas, 2003; Kojic and Darouiche, 2004). Flow is also closely linked to the movement of oxygen and nutrients, both needed for biofilm to grow. *Candida albicans*, a yeast species that typically forms biofilms, has been intensively investigated by medical experts since it is the third most prevalent cause of intravascular infections (catheters; Desai et al., 2014; Herwald and Kumamoto, 2014). However, several more such as *Candida tropicalis*, *Candida krusei*, *Candida glabrata*, and *Candida parapsilosis* biofilm-forming species that damage the human body have been found in recent years (Chandra et al., 2012). *Malassezia* spp. (Velegraki et al., 2015), *Pneumocystis* spp. (Cushion et al., 2009), *Histoplasma capsulatum* (Carreto-Binaghi et al., 2015), *Cryptococcus neoformans* (Martinez and Casadevall, 2015), and *Cryptococcus gatti* are also fungi that can make people severely ill. It was assumed that bacterial and fungal biofilm development followed similar sequences for a long time. However, new insights into the genetic dynamics and the interactions of fungus, hosts, and the environment have shifted this paradigm. Although all fungi go through the same general stages of attachment, adhesion, development, and dissemination, the kinetics of this process vary depending on the species.

### 2.3. Biomedical biofilm

Each year, 4.1 million people in the European Union contract healthcare-associated infections (HAIs) or nosocomial infections, with the cost of treating these diseases being around €5.48 billion. The gravity of the situation is indicated by the number of deaths caused directly by HAIs, estimated to be at least 37,000 every year, and an additional 110,000 deaths caused by other disorders that become complicated by HAIs. Biofilms are responsible for more than 65 percent of hospital infections, according to the Centers for Disease Control and Prevention in the United States, with biofilm-invaded prostheses and indwelling medical devices accounting for a significant portion of these infections (Mah and O’Toole, 2001). Current medical practices employ a lot of implantable medical devices, such as catheters, mechanical heart valves, cardiac pacemakers, coronary stents, cerebrospinal fluid shunts, joint prostheses, orthopedic fixation devices, biliary tract stents, breast implants, contact lenses, dental prostheses, dental implants, and so on. When pathogenic biofilms grow on the surfaces of these devices, they pose a severe risk to public health.

#### 2.3.1. Biofilm infection associated with medical device

In the advancement of medical technology, medical devices, e.g., central venous catheters, peritoneal dialysis catheters, urinary catheters, contact lenses, etc. have become widely used and crucial for clinical treatments. The application of medical devices is sometimes met with infection caused by microbes that detach from biofilms on the medical device (Donlan, 2001). In 1985, urinary catheter-associated biofilms were discovered, and antibiotic resistance in the biofilm was documented (Nickel et al., 1985). Catheter-associated urinary tract infections are quite prevalent, and several studies have investigated them. Biofilm-associated microorganisms present on the inner surface of catheters are antibiotic-resistant and cause chronic disease in individuals due to long-term catheterization (Delcaru et al., 2016). It has been demonstrated that biofilm growth on urinary catheters happens mostly through either colonization of the catheter’s outer surface by direct inoculation during catheterization or migration through the surrounding mucous sheath. Most urinary catheter-associated biofilms are caused by extraluminal microbial invasion, particularly in female patients (Delcaru et al., 2016). Microorganisms can enter the catheter *via* an intraluminal pathway and create a biofilm if a closed drainage system is not maintained or a collecting bag is polluted (Nickel and Costerton, 1992). Furthermore, the microbes can enter the urinary tract and develop a catheter-associated biofilm *via* a bloodstream infection. However, a urinary tract infection is more commonly the cause of sepsis.

Besides catheter-associated biofilms, implanted material-associated biofilms, such as biofilms associated with contact lenses, orthodontal prosthetics, endotracheal tubes, needleless connectors, central venous catheters, intrauterine devices, cardiovascular valves, pacemakers, prosthetic joints, and breast implants, were extensively studied in subsequent studies (Stewart and Bjarnsholt, 2020; Walker et al., 2020). A biofilm is expected to be a key pathogenetic element in the recurrence or persistence of peritonitis through infection or colonization in chronic peritoneal dialysis catheters. Catheter-associated bloodstream infection is a leading cause of nosocomial infections, with high morbidity and mortality (Bouza et al., 2014). Microbial contamination during surgery and catheter implantation may cause biofilm growth on a catheter. These biofilms grow on the catheter’s outer surface. *Staphylococcus epidermidis*, *S. aureus*, and *C. albicans* are prevalent skin microorganisms; hence, they are the most crucial pathogenic causes of catheter-related biofilm infections (Septimus and Schweizer, 2016).

Furthermore, biofilms in the catheter lumen might be caused by bacteremia. Biofilm cells release fewer proinflammatory factors than planktonic cells, which generally trigger significant host responses. In endotracheal tubes, several microorganisms can colonize and produce biofilms. Endotracheal biofilms have been linked to ventilator-associated pneumonia, one of the most common diseases and a major cause of death in intensive care units (Fernández-Barat and Torres, 2016). Biofilm deposition on long-term medical implants, such as prosthetic joints, pacemakers, heart valves, contact lenses, and breast implants, appears to be a primary cause of postoperative problems. Infections can induce inflammation and tissue deterioration around implants, and these infections can occasionally be fatal. Because removing biofilms is challenging, implant replacement should be considered in many patients (Arciola et al., 2018). Biofilms connected with medical devices are the most common source of nosocomial infections. Most biofilm investigations conducted with significant opportunistic pathogens have been extensive. Other nosocomial opportunistic microorganisms forming medical-device-associated biofilms include *P. aeruginosa*, *Escherichia coli*, *Klebsiella pneumoniae*, *A. baumannii*, and *C. albicans*. Most of these infections are resistant to multiple drugs, which makes it very challenging to treat these biofilms (Zhang et al., 2020).

#### 2.3.2. Biofilm infection associated with tissue

Microorganisms can attach to biotic surfaces and spread to other parts of the host organism *via* biofilms, such as epidermal cells and teeth, or they can be found in tissues, such as mucus on mucosal membranes or inside chronic wounds (Zhang et al., 2020). Biofilms generated in gingival crevices and on tooth surfaces are thought to be fundamental causes of gingivitis and periodontitis pathogenesis. They may be linked to the synergistic action of polymicrobes and dysbiosis. Persistent inflammation and chronic infections are linked to an increased risk of cancer (Groeger and Meyle, 2019). The oral microbiome contains up to 750 different microorganisms, including viruses, protozoa, archaea, fungus, and bacteria. Multiple species, such as *Streptococcus* and *Actinomyces*, commonly produce oral biofilms. Tooth surface biofilms can cause dental caries, while supra-and subgingival biofilms underneath and along the gingival area can cause periodontal diseases (Mosaddad et al., 2019). It has been discovered that biofilm production can cause various gastrointestinal illnesses. *Helicobacter pylori* biofilm development on human stomach mucosa has been seen in endoscopically guided biopsies using scanning electron microscopy. Because of biofilm formation, it is difficult to eliminate an *H. pylori* infection (Carron et al., 2006). *Salmonella* can develop biofilms on human gallstones, and bile can dramatically boost *Salmonella* biofilm formation. *Salmonella* biofilm on gallstones may be a source of persistent infection and is linked to an increased risk of gallbladder cancer. Many bacteria, including *E. coli*, *Vibrio cholerae*, and *Salmonella enterica*, can produce biofilms in the host intestines (Mohanta et al., 2020). The bacterial biofilm communities in female vaginal flora are aggressive and loosely tissue-adherent (Davies, 2003). Probiotics, which are live bacteria and yeasts that help maintain gut health and treat and prevent diarrheal disorders, can also produce biofilms. On the contrary, efficient biofilm development of commensal/probiotic-type bacteria has been proven to protect the host against pathogens and lower the prevalence and severity of enterocolitis (Olson et al., 2016).

## 3. Biofilm resistance to antimicrobial agents-mechanisms

Due to resistance to numerous biocides and antibiotics, biofilms are difficult to regulate and eventually remove. Microorganisms re-suspended from biofilms are significantly more resistant than planktonic cells, whereas cells inside biofilms are more resistant than those re-suspended from biofilms. Biofilm cells are hundreds of times more resistant to antibacterial drugs than planktonic cells (up to a thousand-fold increase; Roy et al., 2018). Biofilms are shelters or physical barriers to shield cells from desiccation, chemical disturbance, invasion by other bacteria, and destruction by immune cells (Yan and Bassler, 2019). There are several methods by which biofilm cells increase antibiotic resistance, and these mechanisms differ from those seen in planktonic cells. Initially, antibiotic penetration into biofilms was thought to be the primary responsibility. However, some antimicrobial drugs, such as ciprofloxacin and fluconazole, continue to penetrate biofilms; but the biofilm size wound not decrease. The size of the matrix mesh is now well known to be substantially larger than the dimensions of antibiotic compounds (Yan et al., 2018). Antimicrobial agents’ ability to penetrate a biofilm is influenced by several factors, including the agent’s concentration, the biofilm’s sorption capacity, and the biofilm’s thickness (Stewart, 2015). Some medicines, such as fluconazole, have less effectiveness in *C. albicans* biofilms when producing an exopolysaccharide matrix (Nett and R Andes, 2015). eDNA can diminish antibiotic action by producing cation-limited circumstances, causing lipopolysaccharide (LPS) modification, and decreasing antibiotic uptake, such as aminoglycosides (Mulcahy et al., 2008). eDNA is considered one of the most critical factors in biofilm resistance to antimicrobial drugs. The mechanism of biofilm resistance to antimicrobial agents is provided in Supplementary File 1.

## 4. Food biofilm

Much evidence and research from the last decade suggest that a large amount of food spoilage occurs due to biofilm formation by microorganisms. These biofilms cause food contamination as they serve as a good site for microbial accumulation and disease transmission – surface adhesion and accumulation of bacteria being the primary stage of infection. As per a survey of 2017, around two billion food-mediated illnesses and one million deaths occur globally. Most pathogens that have long-term persistence in food processing units usually grow in biofilms rather than in the planktonic mode – the former being more resistant to antibiotics than the latter (Bai et al., 2021). As such, biofilms by food-borne pathogens like *Clostridium* spp.*, Escherichia coli*, *Listeria monocytogens*, *Pseudomonas* spp., *Staphylococcus aureus*, *Bacillus cereus, Micrococcus* spp. etc., are a huge menace and source of concern for the food industry (Khiralla and El-Deeb, 2015). According to a report from 2016, around 8% of the total deaths caused in Europe that year were due to listeriosis that resulted from cross-contamination between different strains of *L. monocytogens* (European Food Safety Authority, 2016). Certain food industries like dairy and confectionary that use numerous structures like pasteurizers, cheese processing units, milk tanks, butter centrifuges, etc. may provide a suitable substrate for the colonization of different species of biofilm-forming bacteria under different temperatures and pH levels like *Pseudomonas, Geobacillus, Aeromonas hydrophila* and many others (Mizan et al., 2015; Galié et al., 2018). As such, the cross-contamination of *Listeria* strains was observed in food products like salmon, cheese, meat, etc., where this bacteria persisted for a long duration in utensils (forming biofilms) and showed high resistance to changes in temperature, pH, and other chemical compounds (European Food Safety Authority, 2016; Martínez-Suárez et al., 2016). Ideally, biofilms are formed by bacteria to tide over stress and other harmful conditions in the environment. As such, biofilms are a highly resistant system as they house a strategy to evade different antimicrobials and secrete different toxins and enzymes to invade cells from which they can derive nutrition for growth and survival (Hetta et al., 2021). It is noteworthy to mention that while food safety and spoilage caused by biofilm-forming pathogens may cause huge losses to the food sector, the growth of some desirable bacteria like *Lactobacillus,* which is a blessing to the food industry, particularly those dealing with fermented food products, is also hindered by the biofilm-producing bacteria (Giaouris et al., 2014). It has been observed that many strong antibiotics, sanitizers, detergents, and other antimicrobials – the use of some of which is limited in the food industry due to safety measures – have failed to get rid of these pathogens. The EPS-rich biofilms are loaded with nutrients and pathogenic bacteria and can contaminate food materials when they come into contact with them. Rather than causing disruption of the EPS matrix, these conventional methods of disinfection tend to have an antimicrobial potential.

Further, this matrix of the biofilm limits or prevents the access of the disinfectants to the pathogenic organism–the biofilm microenvironment is capable of deactivating foreign chemicals due to their low pH and active enzymatic activity (Han et al., 2017; Barros and Casey, 2020). As such, a more robust and effective technique is needed to eliminate biofilm-forming pathogens, particularly food-borne ones. Nanotechnology, a field that is always changing, could be the answer to this problem and a big help to the food industry.

## 5. Anti-biofilm activity of nanomaterials

**Figure 2:**
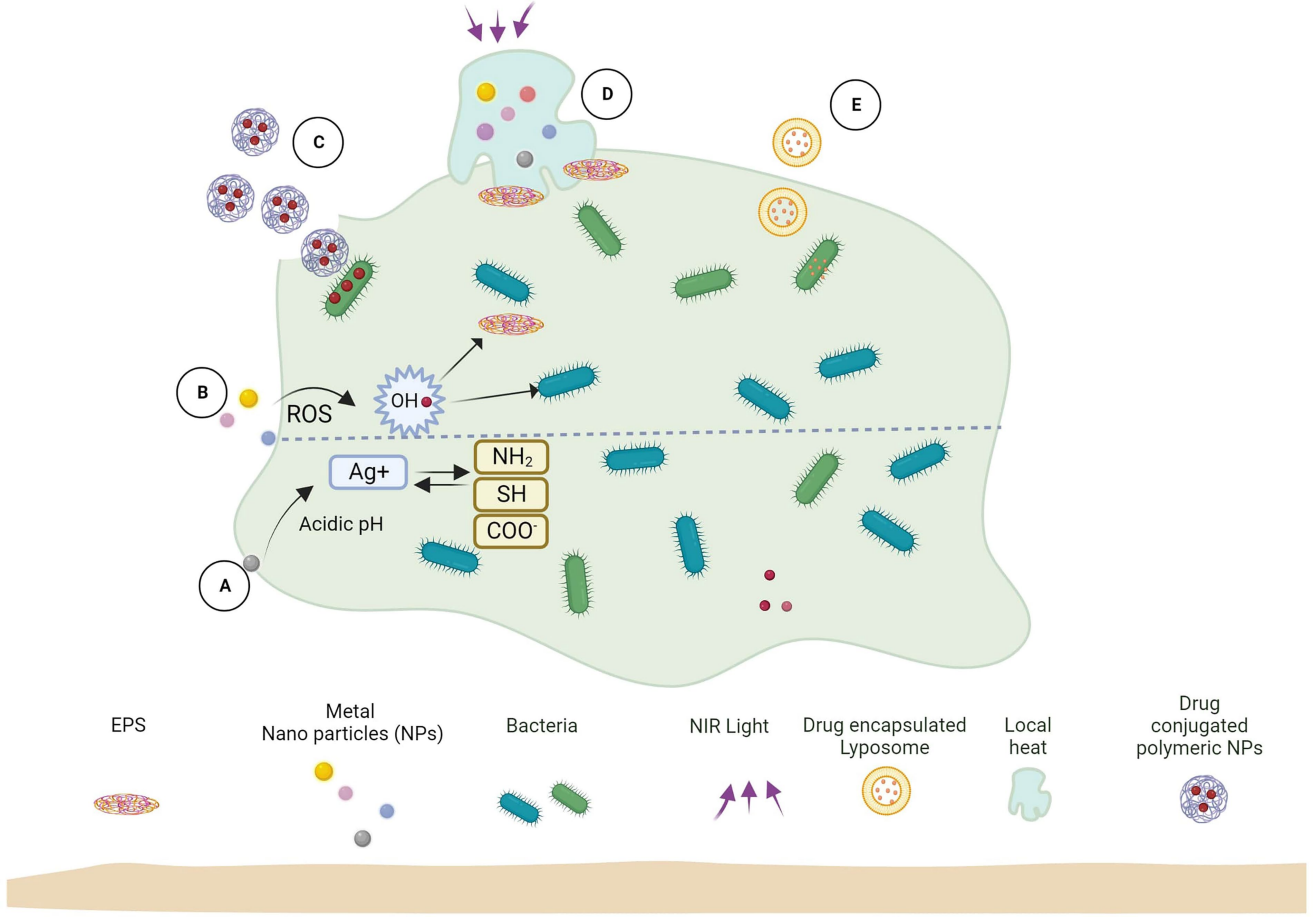
Biofilm and nanoparticle interaction mechanisms. **(A)** Released ions interact with biofilm functional groups. **(B)** Release ROS which kills bacteria and EPS. **(C)** Antimicrobial-loaded polymeric NPs penetrate biofilm and deliver drugs to bacteria. **(D)** Near-infrared (NIR) light irradiation causes localized heat to rise, which kills EPS and bacterial cells. **(E)** Antimicrobial-encapsulated liposomes fuse with bacterial cell membranes to deliver the drug inside.

Nanoparticles and other associated nanomaterials represent promising platforms for establishing new and advanced anti-biofilm technologies. Due to their high surface area to volume area, these materials can easily penetrate different surfaces, including the EPS matrix of biofilms. Though the antimicrobial effect of different nanomaterials has been extensively studied, the EPS matrix of biofilms complicates the action of nanomaterials for such bacteria – different phenomena like NP adsorption and diffusion need to be emphasized (Figure 2).

Different metals and their oxides can survive the harsh and insensitive conditions of various procedures; many (e.g., ZnO) have the advantage of being stable, non-toxic, and relatively safe (Kakati et al., 2020). Nanomaterials synthesized from such metallic oxides have a wide range of applications starting from being antimicrobial, antidiabetic, and finding applications in different sectors like agriculture, waste management, energy and others (Castiglioni et al., 2017; Ameen et al., 2021; Kumar et al., 2022). Oxide nanoparticles of Zn have shown antibiofilm properties on biofilms produced by food-borne pathogens like *Staphylococcus aureus, Staphylococcus enterica*, and *Escherichia coli* in a dose-dependent manner. Ideally, most biofilm inhibitory drugs do not kill bacterial cells but rather alter the structural and physicochemical properties like surface adhesion, hydrophobicity, cell motility, phagocytosis, and reactive oxygen species (ROS) generation. It has been found that the ZnO NPs also show a similar mechanism for biofilm inhibition (Hayat et al., 2021). The anti-adherence and anti-biofilm properties of ZnO NPs against methicillin-resistant *S. aureus* (MRSA) were found to be higher than the antibiotic vancomycin even at low concentrations (Kemung et al., 2020). Nanoparticles composed of CuO also inhibited biofilm formation in strains like MRSA and *E. coli* in a dose-dependent manner. The metal Cu initially targets and destroys the cell envelope of the bacteria and subsequently binds to the genetic material of the microorganism, and generates free radicals that ultimately lead to cell death. The high antibacterial effect of CuO enables these NPs to have a greater anti-biofilm potential than Fe_2_O_3_ NPs (Agarwala et al., 2014). The inherent antibacterial activity of Ag requires no special mention. Like other NPs, AgNPs deposit on the microbial cell wall, inactivate their essential enzymes, and cause toxicity by generating ROS species like hydrogen peroxide and others. These oxides are indeed responsible for the bactericidal effect of AgNPs. It is believed that the bactericidal property of AgNPs allows them to disrupt biofilms formed by notorious pathogens like *Pseudomonas aeruginosa*, *Staphylococcus epidermidis*, and even MDR strains like *Klebsiella pneumonia* (Kalishwaralal et al., 2010; Siddique et al., 2020). It is necessary that anti-biofilm nanoparticles are subjected to clinical trials and undergo proper inspection before making them available. Table 1 shows examples of preclinical studies that show how nanomaterials can stop biofilms from forming.

**Table 1:** Pre-clinical studies of anti-biofilm nanomaterials.

Sl. no.	Anti-biofilm setup	Compound name	Target organisms	MIC (μg/mL)	MBC (μg/mL)	References
1	Antibiofilm activity on micrititre well	AgSiO2 nanoparticles	Staphylococcus aureus	not available	Not available	Geissel et al. (2022)
2	Well diffusion assay	Ag nanoparticles	*B. Cereus,* *S.aureus* *E. coli* *S. Typhimurium*	6.25 μg/mL 100	Not available	Erci and Torlak (2019)
3	Antibiofilm	ZnO-NPs, CuO-NPs	*Strep. oralis*	50 μg/mL	Not available	
4	Tissue culture plate method	AgNPs	*P. aeruginosa* *S. flexneri* *S. aureus* *S. pneumoniae*	0.59 μg/mL 0.60 0.75 0.76	0.7 μg/mL 0.7 0.75 0.75	Gurunathan et al. (2014)
5	Hydrophobicity index	NsEO-AuNPs	*S. aureus* *V. harveyi*	10 μg/mL	Not available	Manju et al. (2016)
6	Antibiofilm activity	Chitosan nanoparticles	*E. faecalis*	5 mg/mL	20 mg/mL	Shrestha et al. (2010)
7	Antibiofilm activity	GNP + proteinase k	*P. fluorescens*	10 μg/mL	Not available	Habimana et al. (2018)
8	Microtiter plate assay	Ginger-AgNPs Chemical-AgNPs	*E. faecalis* -	1/8 (79.53%) 1/8 (87.48%)	Not available	Swidan et al. (2022)
9	Microtiter plates	AgNPs	*S. aureus* *P. aeruginosa* *E. coli*	70 μg/mL 100 80	Not available	Mohanta et al. (2020)
10	Microdilution methods	AgNPs	*P. aeruginosa, E. coli*	50 and 100	100 and 200 μg/mL	Singh et al. (2018)
11	Antibiofilm	AuNPs	*S. aureus* *P. aeruginosa*	0.01–2 mg/mL	Not available	Sathyanarayanan et al. (2013)
12	Polystyrene plate method	ZnO NPs	*P. aeruginosa, S. aureus*	50 and 50	Not available	Doğan and Kocabaş (2020)
13	Microtiter plate assay	AgNPs	Klebsiella pneumoniae	62.5 and 125 μg/mL	250 and 500 μg/mL	Siddique et al. (2020)
14	Polystyrene, glass and acrylic dentures	ZnO NPs	*S. oralis*, *P. aeruginosa, E.* 568 *coli* O157:H7, MRSA	100 mg/mL	Not available	Lee et al. (2014)
15	Microtiter plate assay	ZnO: MgO NPs	*Bacillus subtilis* and *P. mirabilis*			
16	Antibiofilm activity on teeth	Zinc-doped copper oxide (Zn: CuO NPs) and CuO NPs	*S. mutans*	88% and 70%	Not available	Eshed et al. (2014)
17	Microtiter plate assay	MgO NPs	*E. coli,* *P. aeruginosa* *S. epidermidis,* S. aureus MRSA	1 mg/mL	1.0 mg/mL 1.2 mg/mL 0.5 mg/mL 0.7 mg/mL 0.7 mg/mL	Nguyen et al. (2018)
18	Microtiter plate assay	TiO2-NPs-Ca	Candida albicans (fluconazole-susceptible) Candida albicans (fluconazole-resitant)	5.14 μg/mL 5.35 μg/mL	Not available	Haghighi et al. (2013)

Although nanoparticles that are created through physical and chemical processes are used extensively, biogenic nanomaterials are quickly catching up to them. In fact, due to their stability, biocompatibility, non-toxicity, and economic feasibility, biogenic nanomaterials are regarded as being significantly more important. This method uses biomolecules like proteins, carbohydrates, enzymes, vitamins, and secondary metabolites like saponins, alkaloids, flavonoids, terpenoids, and others as reduction and stabilizing agents (Sahayaraj and Rajesh, 2011; Bazana et al., 2019; Khan et al., 2021). Green synthesized AgNPs stabilized by starch have been found to impede biofilm formation by food-borne pathogens like *Shigella flexneri, Salmonella typhi*, and *Mycobacterium smegmatis*, and are non-toxic to macrophages. Interestingly, these AgNPs were more potent as anti-biofilm agents than human cationic antimicrobial peptide, LL-37 (Mohanty et al., 2017). Even green synthesized ZnO NPs using leaf extracts of *Costus igneus* showed dose-dependent anti-biofilm activity against food-borne pathogens *Streptococcus mutans*, *Lysinibacillus fusiformis* (Gram-positive bacteria), *Proteus Vulgaris*, and *Vibrio parahaemolyticus* (Gram-negative bacteria; Vinotha et al., 2019). It is interesting to note that ZnO is one of the metal oxides that has been listed as “Generally Recognized As Safe (GRAS)” by the Food and Drug Administration (FDA) in the United States due to its low toxicity. As such, it has also been incorporated with different plant extracts and has numerous applications in various sectors (Kairyte et al., 2013; Agarwal et al., 2017; Verma et al., 2019). A no. of such nanomaterials that have anti-biofilm potential have been approved clinically and marketed too (Table 2).

**Table 2:** Clinically approved nanomaterials with anti-biofilm efficacy.

Name/Category of the nano-biofilm	Uses	References
Liposomal NO	For treatment of chronic rhinosinusitis (CRS)	Jardeleza et al. (2015)
Liposomal ciprofloxacin and amikacin	For treatment of chronic lung infection in *CF*	Clancy et al. (2013)
AmBisome	Antifungal agent	Gao et al. (2011)
Liposomal Amikacin (Arikace™) and Ciprofloxacin (Lipoquin™, Pulmaquin)	For the therapy of *P. aeruginosa* biofilm infections	Haworth et al. (2019)
Doxil (Sequus) and DaunoXome (Gilead, Nexstar)	Anticancer drugs	Wagner and Vorauer-Uhl (2011)
Nanosilver fluoride to prevent dental biofilms growth (NCT01950546)^#^	To control the growth of *S. mutans* present in dental plaque of children	Pircalabioru and Chifiriuc (2020)
NCT03666195^#^	For treatment of *Candida* infection	Pircalabioru and Chifiriuc (2020)
NCT00337714^#^	For treatment of Central venous catheter (CVC) related infections	Pircalabioru and Chifiriuc (2020)
NCT03752424^#^	For treatment of Foot Infection	Pircalabioru and Chifiriuc (2020)
NCT03554876^#^	For treatment of Peri-implantitis infections	Pircalabioru and Chifiriuc (2020)
NCT00621114^#^	For treatment of Catheter-related bloodstream infections	Pircalabioru and Chifiriuc (2020)

#: Adapted from Table 2 of Pircalabioru and Chifiriuc (2020).

## 6. Biofilm formation and quorum sensing: Nanotechnology as a solution?

Spoilage of food by bacteria is often attributed to a system of communication between bacterial cells called quorum sensing (QS). Food industries are often seen as falling prey to this mechanism, which amounts to large amounts of food spoilage and subsequent economic loss. Cell-to-cell communication between bacteria or QS of food-borne pathogens is mediated by small signaling agents, auto-inducers, which can facilitate bacterial communication both in an intra-and inter-specific manner. QS plays a crucial role in the development of biofilms from bacterial adhesion to full maturity. Several lipolytic, proteolytic, and pectinolytic enzymes are produced during QS, leading to food damage and several diseases (Skandamis and Nychas, 2012; Abudoleh and Mahasneh, 2017; Asfour, 2018). Hence, the disruption of this QS-channel can be considered of prime importance to controlling microbial spoilage of foods and other food-related infections. As such, there is an urgent need to understand the underlying mechanism and signaling molecules involved in QS so that novel strategies and safe tools can be devised and designed to break down this communication between bacteria in the biofilm. Next-generation food preservatives and antimicrobial agents interfere with the QS mechanism and subsequently hinder biofilm formation (Njoroge and Sperandio, 2009; Jordana-Lluch et al., 2020).

Using indicator strain *Chromobacterium violaceum*, it was found that ZnO NPs synthesized with extracts of *Nigella sativa* were able to disrupt the QS mechanism of food pathogens, *E. coli, L. monocytogens*, and *P. aeruginosa* in a dose-dependent manner. Further, these nanoparticles also inhibited the formation of the EPS matrix, leading to biofilm formation suppression. Interference with the EPS synthesis by NPS will hinder the formation, architecture, and stabilization of the biofilms; as a result, the bacteria present in the biofilm can be targeted easily by the antibiotics, and their drug resistance will be subsequently reduced (Al-shabib et al., 2016). Although a lot of critical investigation is still required to understand the anti-biofilm mechanism of NPs, some studies have attempted to understand this concept. Biofilm formation by food-borne pathogens requires pyocyanin and a response regulator, *CzcR*. In Lee et al. (2014) reported that ZnO NPs inhibited pyocyanin production and biofilm formation by the *CzcR* mechanism. Further, it also repressed the QS by lowering the production of autoinducers. As a result, the biofilm’s attachment to the surface was disrupted completely (Lee et al., 2014). Due to the breakdown of the QS interaction between the bacteria, the maturation of the biofilm was also significantly impaired. AgNPs exhibited a similar mechanism on biofilms produced by *P. aeruginosa* (Glišić et al., 2016). Functionalized chitosan nanoparticles are usually loaded with DNAse and oxacillin. This composite can decrease biofilm formation and width (~99%) in nearly all strains of *S. aureus* and *P. aeruginosa*. These NPs, when conjugated with natural phytochemicals like flavonoids or alkaloids, have been found to decrease or inhibit QS between bacteria. Nanocomposites interact with bacteria *via* electrostatic interaction and deliver the quorum-quenching compound to the bacteria – this electrostatic adsorption is believed to be one of the underlying mechanisms of NPs to inhibit QS by food-borne pathogens (Sun et al., 2019; Nag et al., 2021). It was also demonstrated that carbon-based nanomaterials acted as QS inhibitors; low concentrations of graphene oxide released from these nanomaterials did not affect the bacteria but inhibited biofilm formation. This oxide molecule adsorbed on protease converted by QS of bacterial pathogens interfered with the QS mechanism and prevented biofilm maturation. However, after some generations, most food-borne pathogens can override this inhibitory effect (≥150). Further, it was also reported that CuO NPs coupled with carbon nanomaterials downregulated gene expression of *Methylobacterium* spp. to inhibit QS and prevent biofilm formation (Seo et al., 2018; Zhang et al., 2018; Cui et al., 2022). Ironically cross-linked NPs tend to be unstable in the colloidal culture media but exhibit high anti-QS activity. Functionalized AgNPs can interact with the bacterial cells and disintegrate them without allowing QS and subsequent biofilm formation.

However, the inhibition of QS alone is insufficient to prevent biofilm formation in the food industry. A holistic strategy has to be designed in this regard. A synergistic anti-biofilm approach was developed that combines antibiotics, QS inhibitors, and photodynamic therapy in the same nanostructure. The NPS carries the drugs and releases the antibiotics and inhibitors sequentially upon interaction with the microorganisms. The inhibitors sensitize the bacteria completely, laying down the foundation for the action of antibiotics to exert their effect. Finally, the combined action of the QS inhibitors and photodynamic therapy can overcome the resistance of the bacteria, cause phenotypic change of the organism and change its hydrophobicity, which subsequently prevents bacterial invasion and biofilm formation (Sun et al., 2019; Cui et al., 2022).

## 7. Anti-biofilm effect of nanoparticles/nanosystem

**Figure 3:**
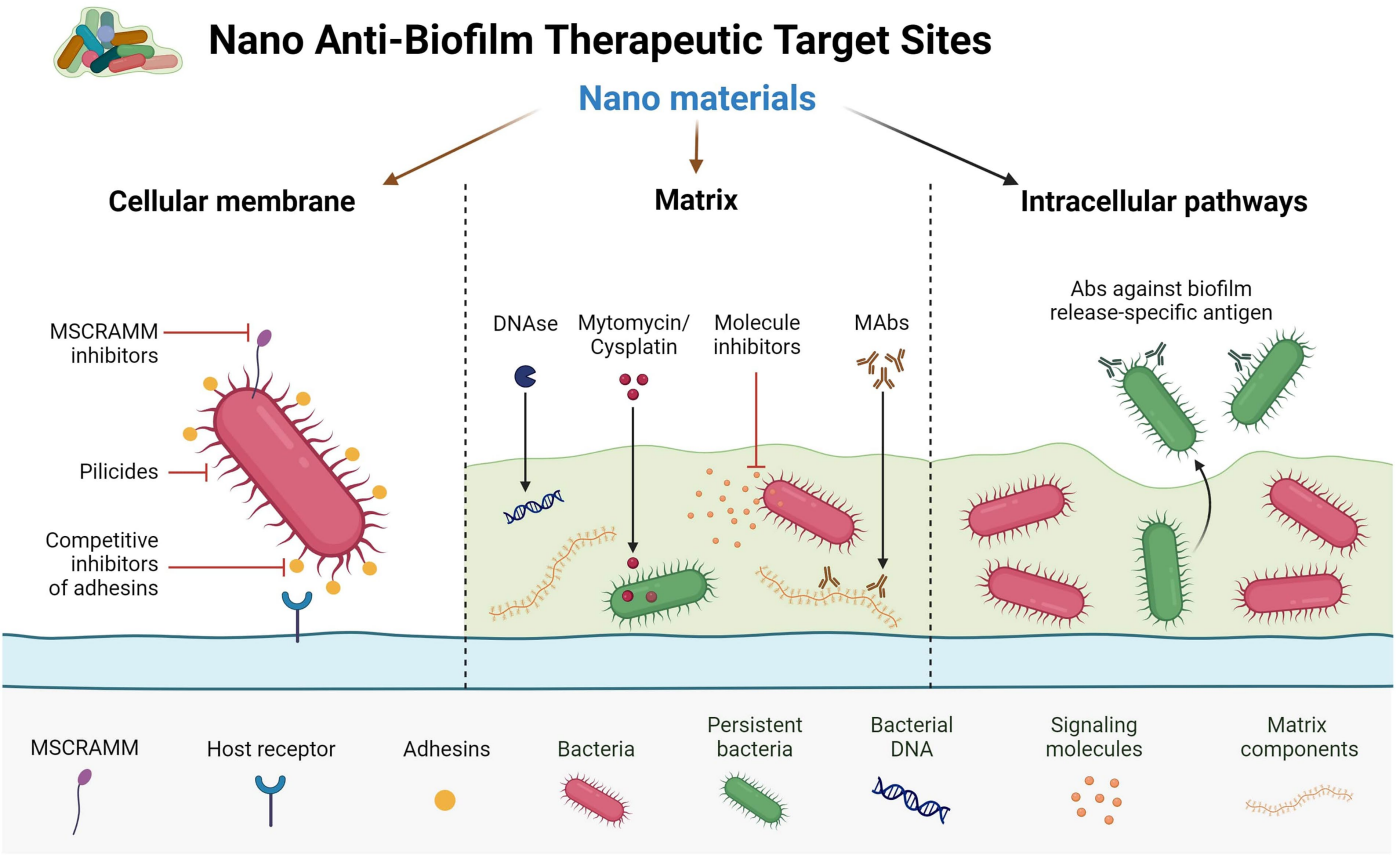
Different therapeutic target sites against biofilm formation by nanomaterials.

Drugs and delivery methods focused on specific targets will be the focus of the next wave of pharmacological innovation. In recent years, new nanotechnologies have been authorized in clinical practice as diagnostic instruments or as precise drug delivery systems for tissues smaller than 100 nanometers. As a result of current therapies’ *in vivo* instability, lack of bioavailability or solubility, minimal absorption, and toxicity, nanoparticles (NPs) are being developed to tackle these issues. Aside from increasing bioavailability *via* specialized absorption mechanisms, these products are designed to enhance the effectiveness and dosage of other FDA-approved medications and even prolong their shelf life beyond expiry. Nanostructures have several benefits in drug administration, including increased tissue penetration, cellular absorption, and reduced adverse effects. The detailed mechanisms of the role of nanomaterials in biofilm inhibition or eradication are well depicted in Figure 3.

### 7.1. Metal nanoparticles

Metallic nanoparticles are a fundamental kind of nanoparticle that has inherent anti-biofilm properties. Surface functional groups or charge contact with the biofilm might cause some metallic nanoparticles to emit toxic ions that attack bacteria or the EPS (Mout et al., 2012; Qayyum and Khan, 2016; Hiebner et al., 2020).

One of the most researched metallic nanomaterials, silver has excellent bactericidal effects on a wide variety of microbe species, and it has been proven to be able to remove biofilms. AgNPs are commonly used as antimicrobial agents and have more potent antibacterial activity than antibiotics. They are also employed in clinically produced implants, catheters, and wound dressings (Fulaz et al., 2019). In addition to being effective antimicrobials, their high surface-to-mass ratio makes them a great candidate for use as monolayers on biomaterial surfaces (Taglietti et al., 2014). An investigation of the antibacterial effects of AgNPs on *Acinetobacter baumannii*, *E. coli*, and *S. aureus* was carried out and took 24 h in biofilm disruption tests; hence AgNPs may destroy biofilms of *A. baumannii, E. coli*, and *S. aureus* to the extent of 88, 67, and 78%, respectively, and a mixture of biofilms to the extent of 64% following a 24-h treatment at 37°C (Salunke et al., 2014). Using *Acinetobacter calcoaceticus* functionalized AgNPs with diameters varying from 4 to 40 nm could destroy the biofilms of 20 distinct harmful bacteria (Gaidhani et al., 2013).

Regarding antimicrobial action, when AgNPs break apart, they release silver (Ag^2+^) ions, which merge with the cell membrane. This depolarizes the cell wall, changing its negative (−ve) charge as well as permeability and killing the bacteria inside. Furthermore, when Ag^2+^ ions enter a bacterium, they trigger a cascade of damaging events, including the oxidation and destruction of cellular constituents, suppression of respiratory chain enzymes, the procreation of reactive oxygen species (ROS), and the subsequent suppression of ATP production and DNA replication (Liu et al., 2019c).

According to LewisOscar et al. (2021), AgNPs suppress the procreation of *P. aeruginosa* biofilm by blocking the generation of rhamnolipids by interfering with the QS system. These also disrupt proteins due to the binding of ionic constituents to cysteine residues, causing more deterioration and impairing the formation of exo-polysaccharides (Rajivgandhi et al., 2021). Ansari et al. (2014) ascribed AgNPs activity inside the biofilm to disperse the water channels throughout the biofilm, permitting nutrient delivery. As a result, AgNPs may gain direct access to the exo-polysaccharides *via* these channels to perform their antimicrobial action.

Nonetheless, certain biofilm bacteria, such as *V. cholerae* and ETEC, may resist AgNPs to some extent (Salem et al., 2015). Despite concerns about the cytotoxicity and genotoxicity of AgNPs, no clinically significant differences in metabolic, urinary, hematologic, or physical measures were noticed, and there were no changes in the production of pro-inflammatory cytokines or reactive oxygen species in the lungs when a colloidal silver nanoproduct available commercially was administered orally (Munger et al., 2015). According to a current study, AgNPs exerted a low level of toxicity *in vivo* that was well tolerated at a modest dose (Hussain et al., 2019). However, AgNPs should be well bound to approve biomaterial surfaces to minimize the danger of potential harmful effects of NPs (Taglietti et al., 2014). At the same time, AgNPs functionalized with other metals/polymers/antibiotics can increase their effectiveness against the biofilm by preventing aggregation, reducing cellular uptake and cytotoxicity, and enhancing site-selective delivery (Liu et al., 2017). Permana et al. (2021) found that poly (−caprolactone) encapsulated AgNPs decorated with chitosan made themselves less toxic and better at getting rid of certain infections.

Compared to silver nanoparticles, gold nanoparticle (AuNPs) are more effective at inhibiting biofilm growth. This is because AuNPs decrease the production of exo-polysaccharides (Rajkumari et al., 2017) and the hydrophobicity index. The breakdown of bacterial membranes and impediments to ATPase synthesis is thought to be the antibacterial mechanism of AuNPs. Additionally, the ribosomal subunit’s ability to bind to tRNA is inhibited, nicotinamide is attacked, and the bacterial respiratory chain is impacted (Baptista et al., 2018). Including antibacterial qualities, AuNPs exhibit photothermal characteristics when exposed to near-infrared (NIR) light. This is because aggregated AuNPs absorb light in a red-shifted manner, which causes a dramatic increase in localized heat. This, in turn, represents yet another efficient lethal factor within the infectious biofilm without affecting encircling tissues, which needed considerably more heat because their cell sizes were more significant than bacterial ones (Liu et al., 2019b; Handa et al., 2022). Without having a substantial impact on cell development, AuNPs’ influence on biofilms may be limited, and AuNPs’ ability to reduce biofilm creation is dose-dependent (Ali et al., 2020). The antibacterial abilities of AuNPs can be improved in several ways, including by combining them with other substances, antibiotics, or even AMPs. In addition, making use of their ability to convert light into heat offers a potential strategy for getting rid of bacterial biofilm infections.

Ramya et al. (2015) studied the synthesis of actinobacterial synthesized selenium nanoparticles for their anti-biofilm activity. They synthesized selenium nanoparticles, extracellular from *Streptomyces minutiscleroticus* M10A62 isolated from a magnetite mine. Pathogenic bacteria may be protected against antibiotics by the production of biofilm, which can lead to the development of long-term illnesses. *Acinetobacter* spp. biofilm development was a major problem in hospital-oriented infections. Biofilm formation in six *Acinetobacter* strains has been thoroughly investigated. When six *Acinetobacter* species were grown *in vitro*, crystal violet was used to test the inhibitory effect of SeNPs on biofilm formation. Spectroscopic observations showed that when the quantity of SeNPs increased, the growth profiles of the six tested bacterial strains tended to decrease. Treatment with nanoparticles reduced biofilm growth to zero after 48 h and increasing the incubation duration had no discernible effect. Concentrations of selenium nanoparticles that prevent maximal biofilm development by six *Acinetobacter* species. In this work, the SeNPs substantially reduced the development of *Acinetobacter* sp. for the first time.

### 7.2. Metal oxide nanoparticles

Zinc oxide nanoparticles (ZnO-NPs) have been explored for their impact on oral cavity bacteria by Tabrez Khan et al. (2013) (*S. oralis* ATCC 35037). ZnO-NPs were created using the sol–gel process, and at a concentration of 100 mg/mL, they prevented the growth of biofilms on polystyrene, glass, and acrylic dentures. These nanoparticles displayed antibacterial and anti-biofilm activity against the oral bacterial isolates of *Rothia dentocariosa* (Ora-7) and *Rothia mucilaginosa* (Ora-16) (Khan et al., 2014). The study results revealed that Zn^2+^ and/or ZnO-NPs have anti-biofilm action against *P. aeruginosa* PAO1, *E. coli* O157:H7 (ATCC 43895), a methicillin-sensitive *S. aureus* (MSSA; ATCC 6538), and a methicillin-resistant *S. aureus* (MRSA; ATCC BA-1707) (Lee et al., 2014).

Titanium dioxide nanoparticles, also known as TiO_2_-NPs, and ethylene diamine tetra acetic acid, often known as EDTA, were investigated by Haghighi et al. (2012) for their potential use as antifungal agents against *C. albicans* biofilms. Ibrahem et al. (2014) used vaginal *Lactobacillus crispatus* isolated from healthy Iraqi women to synthesize titanium nanoparticles (TiNPs). The nanoparticles were tested for antibacterial and anti-adhesive properties against *E. coli*, *K. pneumoniae*, *Morganella morganii*, *A. baumannii*, and *S. aureus* isolated from urine samples of Iraqi women suffering from recurrent urinary infections. The TiNPs displayed an anti-adhesive effect against all clinical isolates (excluding *E. coli*), with the highest and lowest efficiency against *M. morganii* (48%) and *A. baumannii* (6%), respectively. Maurer-Jones et al. (2013) evaluated several different types of *in vitro* synthesized TiO_2_-NPs and commercially available Evonik Aeroxide Degussa P25 and Eusolex T-Eco, for their ability to suppress *Shewanella oneidensis* biofilm by using quartz crystal microbalance and riboflavin secret.

Catalytic iron oxide nanoparticles (CAT-NP) were activated by the acidic pH of the biofilm matrix and have been used for their antibacterial qualities for decades, with the most often used metal-based nanoparticles being iron oxide-based and copper oxide-based nanoparticles (Naha et al., 2019). Using mixed metal oxides in precise ratios may have a more significant impact than utilizing a free metal oxide, such as ZnO: MgO NPs, which at low concentrations prevent Bacillus subtilis and *P. mirabilis* from forming biofilms. According to Eshed et al. (2014), the zinc-doped copper oxide (Zn: CuO NPs) coated teeth improved the killing of *S. mutans* and lowered biofilm development by 88 percent as opposed to 70 percent with free CuO NPs.

**Figure 4:**
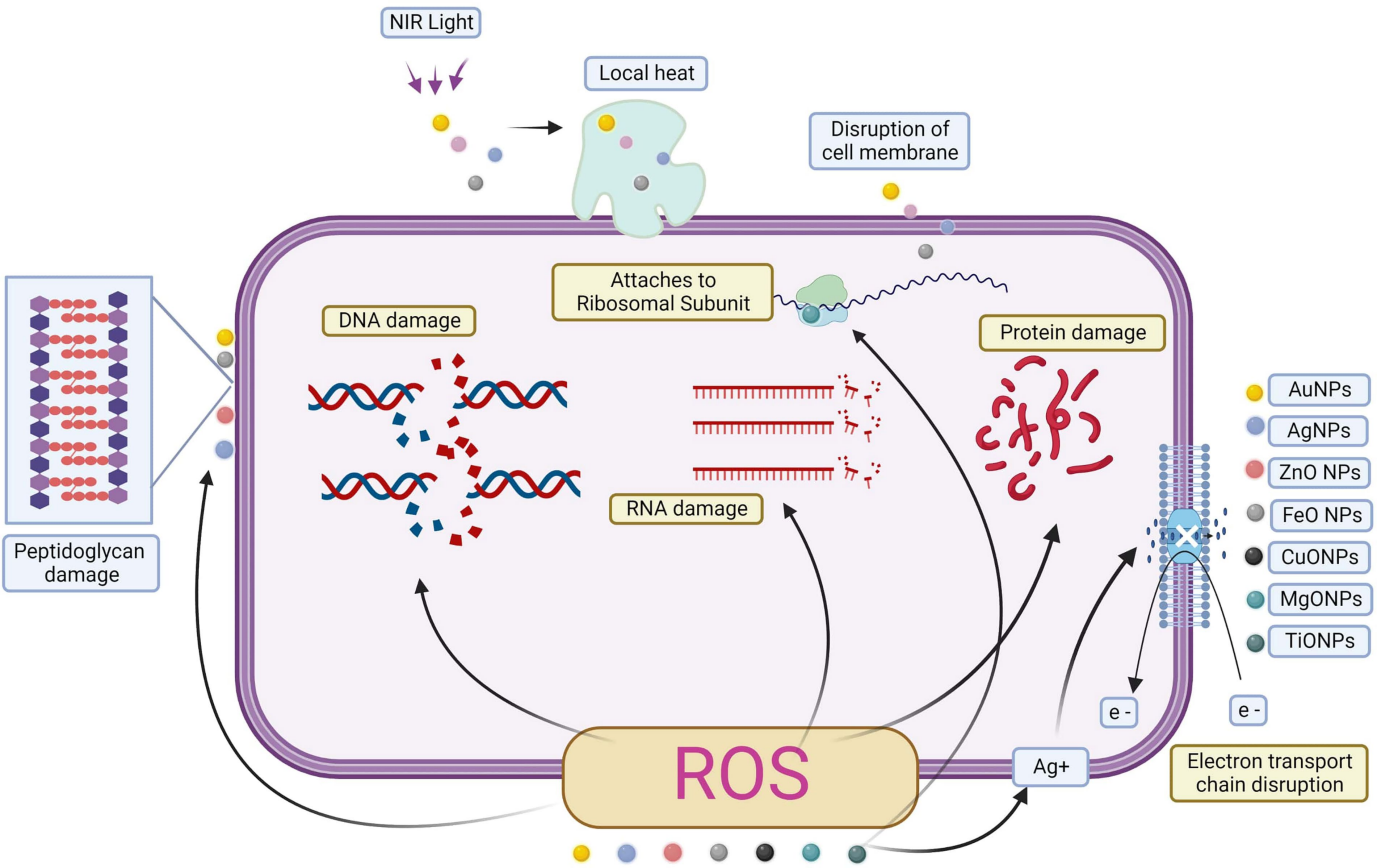
Metal and metal oxide nanoparticles in biofilm activity.

Antibacterial properties of metal or metal-oxide nanoparticles may take several forms (Figure 4). Biofilm development is inhibited by a variety of mechanisms, including direct contact with the bacterial cell wall, glucan synthesis suppression, immune cell recruitment, the creation of reactive oxygen species (ROS), and harmful interactions with bacterial DNA and proteins (Aderibigbe, 2017; AlMatar et al., 2017; Hemeg, 2017). All of these pathways have high bactericidal action, even against persistent latent cells resistant to conventional antibiotics (Bassegoda et al., 2018).

CAT-NP50 showed early anti-biofilm activity, and these nanoparticles generated reactive oxygen species when combined with hydrogen peroxide (Gao et al., 2016). ROS-mediated oxidative stress often results in biomolecule oxidation and cell component damage (Li et al., 2012; Dwivedi et al., 2014). When ROS was present, the biofilm exopolysaccharide matrix degraded and *S. mutans* was killed. In particular, *S. mutans*-produced iron oxide particles showed strong peroxidase-like activity only at an acidic pH. Importantly, normal tissues were shielded from off-target effects due to the nanoparticles’ pH-responsive nature, which restricted free radical formation under physiological settings. When applied to a biofilm model for rats, the iron oxide particles had poor colloidal stability and indiscriminate tissue adhesion, which hindered their capacity to be applied to humans. That means that to keep the iron oxide catalytic properties intact and promote the nanoparticle adhesion to biofilms rather than gingival tissue, dextran coatings were designed for CAT-NPs, known as Dex-NZM in this issue. Treatments *in vivo* decreased caries incidence and severity like that of bare iron oxide particles (Naha et al., 2019). Due to the transfer of several antimicrobial medicines into the biofilm *via* a magnetic field, Fe_3_O_4_ NPs have a less substantial antibacterial property but a significant antibiofilm potential (Hornby et al., 2001).

At sufficiently high particle concentrations (on average 100–1,200 g/mL), MgO NPs exhibit activity against Gram-positive and Gram-negative bacteria, spores, and viruses (Pitangui et al., 2012). In particular, the minimum inhibitory concentration (MIC) of MgO NPs for *E. coli* was 1 mg/mL (Nguyen et al., 2018). Recent research has demonstrated that nanoparticles of magnesium oxide are effective in combating biofilms produced by *E. coli* (250 g/mL), *K. pneumoniae* (125 g/mL), and *Staphylococcus aureus* (500 g/mL). *Escherichia coli*, *S. aureus*, and *K. pneumonia* were incubated with MgO for 12 h, and as a result, the bacteria’s adherence to the plastic’s surface decreased significantly. This prevented the formation of biofilm. It was also possible to determine the effect of MgO on mature biofilms. When treating biofilms with a sub-inhibitory concentration of 0.5 MIC, there was a considerable decrease in the biofilm’s biomass (Hayat et al., 2018). Another recent study found that the production of *S. aureus* biofilms is substantially more difficult at a concentration of 10 g/mL, which was reported (Wong et al., 2020). At a size of 8 nm, MgO NPs exhibited a robust inhibitory effect on the formation of biofilms by *E. coli* and *S. aureus* (Makhluf et al., 2005). *Ralstonia solanacearum* stopped growing in biofilms, and the production of biofilms decreased as more bulk MgO treatments were used. Therefore, the antibiofilm capabilities of MgO NPs are considerable, although significant impacts only occur at sufficiently high particle concentrations.

The Al_2_O_3_ NPs are toxic to bacteria in both their planktonic and biofilm forms, but plankton cells are more vulnerable to Al_2_O_3_ NPs than biofilms (Chrzanowska and Załęska-Radziwiłł, 2014). The *P. aeruginosa* biofilm was shown to have a minimum inhibitory concentration of 1.6–3.2 mg/mL. Clinical isolates of *P. aeruginosa* with extended-spectrum b-lactamases and Metallo-b-lactamases were treated at 2 mg/mL and experienced complete growth suppression (Ansari et al., 2015). Al_2_O_3_ has weaker antibacterial and anti-biofilm capabilities than other oxides. Thus, it can only be combined with biocides and function in nanocomposites. The information presented above shows that metal oxide NPs can be effective materials for combating biofilms. The degree to which a particle has anti-biofilm qualities is directly related to its effectiveness against bacteria. These characteristics are mostly influenced by the synthesis process, the size, and the shape of the particles. The table summarizes the results of several studies related to the application of metals and metal oxide nanoparticles used for the control of microbial biofilms is available in Supplementary File 2.

It is possible that other nanomaterials, such as carbon-based compounds for usage as drug nano-carriers, would be suggested for biological uses. Graphene (G) and graphene oxide (GO) are examples of carbon compounds that have been shown to be highly effective in transporting metals or metal oxides such as silver nanoparticles (AgNPs) and copper oxide nanoparticles (CuOxNp) (CuONPs). To create GO, a small number of carbon atom layers are packed into a honeycomb-like 2D lattice (Kumar et al., 2019; Li X. et al., 2019). Many researchers have been interested in it over the last few decades because of its impressive features (Pulingam et al., 2020; Zou et al., 2020). These include a high specific surface area, excellent thermal stability, strong biocompatibility, planar behavior, and remarkable electrical properties. There are many oxygen-containing functional groups in GO, including carboxyl, hydroxyl, and epoxy groups. Inducing reactive oxygen species (ROS; Karthik et al., 2020; Menazea et al., 2020) is made possible by the electron-generating potential of these functional groups on GO. Further, its excellent physical and chemical characteristics (Fujii et al., 2020; Mirmohseni et al., 2020) allow it to exhibit antibacterial activity. Chemical contact *via* the oxidative process or by electron transition between GO and pathogen (ROS; Dat et al., 2020); direct touch between GO nanosheets and bacterial cells, which may induce membrane collapse. Many researchers have proposed using GO for various medicinal purposes, including as disinfection (Ahmad et al., 2020; Alipour and Namazi, 2020). The hydrophilic properties of GO suggest it might serve as a fantastic platform for the delivery of various compositions across the biological environment (Alsaedi et al., 2019). Many studies have looked at the potential uses of metal oxides like CuONPs, one of which is as an antibacterial agent in biopolymer-based materials. Antibacterial activity of cellulose/GO/CuO nanocomposite films was studied, for instance, by Xie et al. The results suggested that a direct contact between GO sharp sheets and CuO nanorods with the cell and lipid membrane of bacteria might be the mechanism of antibacterial action (Ahmed et al., 2019; Xie et al., 2020). This contact would result in a change in membrane permeability, killing off the bacteria.

Scientists used a laser ablation method to examine the matrix with silver and copper oxide nanoparticles embedded through GO (Menazea and Ahmed, 2020). The diameters of the produced AgNPs ranged from 5 to 30 nm, whereas those of the CuONPs were between 3 and 10 nm. Each one was dispersed among GO nanosheets separately. In tests using the HFB4 cell line, the cell viability of various mixtures showed that the proportion of living cells was not affected (84.7 3.8%). However, the photocatalytic activity of AgNPs was studied for its potential antibacterial effects against *E. coli* and *S. aureus*, with results showing an inhibition zone of about 10.2 mm for the former and 15.2 mm for the latter in the case of AgNPs@GO. The incorporation of metallic and metallic oxide nanoparticles into GO nanosheets to boost antibacterial activity provides further evidence that biocomposites can be optimized for specific clinical applications, such as antimicrobial and disinfectant strategies. Scientists have developed a novel, simple method to construct stable rGO-Cu_2_O nanocomposites by exploiting the electrostatic contact and unique electronic transition between rGO and Cu_2_O (Yang et al., 2019). These nanocomposites exhibit potent antibacterial ability and sustained efficacy. This allowed the 25 nm monodispersed Cu_2_O nanoparticles to be evenly distributed throughout the rGO nanosheet surface. Antibacterial tests showed that rGO-Cu_2_O nanocomposites exhibited potent, sustained antibacterial efficacy against *E. coli* and *S. aureus*. The stability test, copper ion release test, and detection of reactive oxygen species all corroborated that the increased antibacterial mechanisms of rGO-Cu_2_O nanocomposites resulted from the additive effects of the nanocomposites’ sustained release of copper ions, elevated ROS production ability, and excellent dispersion.

The antibacterial activity of AgNPs, GO, and Ag-GO nanocomposites was investigated, and it was found to be most effective against gram-negative bacteria (*E. coli*) than against gram-positive bacteria (*S. aureus* and *S. epidermidis*) and yeast (*C. albicans*; Yang et al., 2019). Inhibition concentrations for all bacteria and yeast strains examined were found to be drastically reduced when GO was decorated with AgNPs, suggesting a synergistic impact. The thickness of the microorganism’s cell wall also affects Ag-antibacterial GO’s activity. It is possible that the increased resistance to GO-antibacterial Ag′s actions in gram-positive bacteria (such *S. aureus* and *S. epidermidis*) is due to their thicker peptidoglycan coating. Damage to the cell occurred as a result of oxidative stress owing to the generation of reactive oxygen species (ROS) and the disruption of membrane functioning induced by a contact between released AgNPs/Ag^2+^ ions and the cell membrane. More research is required to elucidate the process by which ROS are generated.

It has become technically feasible to further enhance the antimicrobial activity by reducing the size of AgNPs to a value comparable to the Fermi wavelength of electrons (1 nm), thereby producing the next generation of silver-based antimicrobial agents known as ultrasmall silver nanoclusters (NCs). This was accomplished by reducing the size of silver nanoparticles to a value comparable to the Fermi wavelength of electrons. Because Ag NCs have a greater surface area to volume ratio than other nanocrystals, they are able to speed up the discharge of Ag^2+^ ions (Wang et al., 2013). Ag NCs are able to internalize the cellular membrane and directly interact with the subcellular organelles of pathogenic microorganisms (Buceta et al., 2015; Neissa et al., 2015). Ag NCs have the same size as subcellular organelles, therefore they are capable of doing this. In addition to this, the local concentration of Ag^2+^ ions that are assembled on the surface of Ag NCs is significantly greater (Yuan et al., 2013, 2014). In general, the attractive physicochemical properties of Ag NCs have the potential to enhance the bactericidal activity of these nanoparticles (Chakraborty et al., 2013).

Physiological circumstances, on the other hand, cause Ag NCs to become reactive and unstable. Oxidation (Ritchie et al., 2007), which led in aggregation and loss of antibacterial action, frequently impedes the practical uses of Ag NCs. This was the cause of the loss of antibacterial activity. To find a solution to this issue, Ag NCs might be engineered to be encased within other drug carriers or to be doped into these carriers so that they maintain their antibacterial effectiveness over time. Because of the remarkable biocompatibility of silica nanoparticles, the Ag NCs-SiO_2_ composite has garnered a significant amount of interest. There are two primary ways that Ag NCs-SiO_2_ antibacterial agents may be designed (Qian et al., 2012). These are the core-shell and surface dispersed architectures. In spite of the fact that the core-shell structure of Ag NCs-SiO_2_ makes it possible for silver ions to be steadily released from the Ag NCs core, the antibacterial capability of the nanocomposites is poor because of the inefficient release of Ag^2+^ ions over the SiO_2_ shell. On the other hand, directly depositing Ag NCs on the surface of silica nanoparticles makes it possible for a high rate of Ag^2+^ ion release. However, the Ag NCs are easily consumed by weak binding or chemical erosion in the biological media (Kumar et al., 2010), which results in a rapid decrease in antibacterial activity. For this reason, it is very necessary to create a novel structure of Ag NCs-SiO_2_ that is capable of the effective release of Ag^2+^ ions over a prolonged period of time. This is necessary so that antibacterial applications may be used in the real world.

Because of their vast surface area, organized pore structure, and high level of biocompatibility, mesoporous silica nanoparticles (MSNs) are becoming more attractive as carriers for the regulated release of a wide variety of therapeutic drugs. MSNs, which were designed by researchers and used as the basis for anchoring Ag NCs, were adopted. A one-pot synthesis was used to generate Ag NCs adorned mesoporous silica nanoparticles, which were referred to as Ag NC-MSNs (Liu J. et al., 2019). The successful prevention of the aggregation of Ag NCs was facilitated by the homogenous dispersion of ultrasmall Ag NCs across the whole framework of MSNs, which also encouraged the continuous discharge of Ag^2+^ ions. Because of the structure of Ag NC-MSNs, Ag NCs that are repositioned on the surface ensure a reasonable Ag^2+^ ion release rate, which in turn results in a high antibacterial activity. On the other hand, Ag NCs that are embedded in the pores of MSNs serve as a reservoir for Ag^2+^, which enables the antibacterial activity to be maintained over time. In addition, the mesoporous structure would make it easier for bacteria to adsorb onto and interact with the silver nanocrystal microscale structures. Similarly, many other researchers studied the effect of nanoparticles on inhibition of bacterial population (Ramasamy et al., 2017, 2020; Raj et al., 2022).

#### 7.2.1. Mechanism of metal ions

Recent research has shown that metal ions can kill bacteria in ways unrelated to hyperosmotic shock. For example, cellular malfunction, enzyme inactivation, and DNA damage occurred when metal ions crossed a hazardous threshold and outcompeted the right metal-ion cofactors naturally present in intracellular proteins (Lemire et al., 2013). Particularly vulnerable to site-specific inactivation by hazardous metals was the bacterial Fe-S dehydratase family, which was inhibited when the Fe2+ cofactor was replaced with exogenous metals from MOFs (Xu and Imlay, 2012). Lysine, proline, histidine, and arginine residues in *E. coli* proteins were shown to be particularly vulnerable to metal-catalyzed oxidation, which then produced carbonyl derivatives that rendered the proteins dysfunctional (Stadtman and Levine, 2003).

Also, studies have shown that Cu^2+^ increases intracellular ROS, notably in *E. coli*, where superoxide (O2) damages DNA and inhibits the dehydratases, such as aconitase B and fumarases A and B, necessary for cell development (Macomber et al., 2007). Exposure of yeast to a toxic dose of Cu^2+,^ on the other hand, resulted in an upregulation of genes encoding reactive oxygen species (ROS)-scavenging enzymes, which protected the yeast from hydrogen peroxide (H_2_O_2_) and oxygen radical (O_2_) toxicity (Yong et al., 2008; Faulkner and Helmann, 2011), demonstrating that such antimicrobial mechanisms can be pathogen-specific. When other metals like Ag^2+^, Co^2+^, and Zn^2+^ bond with the sulfur of cellular thiols (such glutathione, a redox mediator in bacterial cells), they cause the thiols to be oxidized and disulfides to be produced (Harrison et al., 2009). Synergistic cytotoxicity is the outcome of an increase in protein oxidation by metal ion-mediated reactive oxygen species (ROS) and a decrease in glutathione (Table 3). Standard electrode potentials were shown to be correlated with the toxicity of redox-active metal ions (Harrison et al., 2007). Co^2+^, an example of an exogenous metal from MOFs, can be genotoxic by altering intracellular iron homeostasis, which increases the Fenton reaction and causes DNA damage and cell death (Touati et al., 1995).

**Table 3:** Antibacterial action of metallic nanoparticles.

NP type	Size (nm)	Strain	References
Ag	17.5	*P. aeruginosa* ATCC 27317	Dorobantu et al. (2015)
	38.8	*S. aureus* ATCC 25923	
	20–25	*A. baumanii* BAA-747, *P. aeruginosa* ATCC 27853	Martinez-Gutierrez et al. (2010)
		*B. subtilis* ATCC 6333	
		*E. coli* ATCC 25922, MRSA ATCC 700698, *M. smegmatis* ATCC 700084	
		*M. bovis* BCG ATCC 35374	
		*S. aureus* ATCC 25923	
	9–21	Nitrifying bacteria	Choi and Hu (2008)
	9–43	*E. coli*	Ivask et al. (2014)
		*E. coli*	
		*E. coli*	
		*E. coli*	
		*E. coli*	
	18	*E. coli*	Cui et al. (2013)
	10	Gram-positive strains and *Bacillus*	El Badawy et al. (2011)
	39	*E. coli* ATCC 10536	Pal et al. (2007)
	5–10	*E. coli* MTCC 405	Aazam and Zaheer (2016)
		*S. aureus* MTCC 3160	
	5–40	*A. punctate*	Sudheer Khan et al. (2011)
		*E. coli* ATCC 13534, *E. coli* ATCC 25922	
		*M. luteus*	
	142	*E. coli* K12 MG 1655	McQuillan et al. (2012)
	5–15	*L. monocytogenes* ISP 6508	Tamayo et al. (2014)
	9.2	*E. coli* K12 MG 1655	Lok et al. (2007)
	35	*A. vinelandii* ATCC 13705	Yang et al. (2013)
		*N. europaea* ATCC 19718	
		*P. stutzeri* ATCC 17588	
	22.5	*E. coli* (clinical isolate)	Shahverdi et al. (2007)
		*S. aureus* (clinical isolate)	
	7.1	*E. coli* MTCC 062	Ramalingam et al. (2016)
		*P. aeruginosa* MTCC 424	
	142	*E. coli* K12 MG 1655	McQuillan and Shaw (2014)
	35.4	*E. coli* K12 ATCC 25404	Xiu et al. (2011)
	30	*E. coli*	Wigginton et al. (2010)
	60	*E. coli* K12 MG 1655	Gou et al. (2010)
	20–30	*P.* ssp. FPC 951	Soni et al. (2014)
	10–20 nm	*S. aureus*, *P. aeruginosa*	Davidović et al. (2019)
	8–16 nm	*S. aureus*, *E. coli*	Chen J. et al. (2020)
	16 nm	*S. aureus*, *E. faecalis*, *P. aeruginosa*, *E. coli*	Konvičková et al. (2019)
	2–10	*K. pneumonia* ATCC 700603	Railean-Plugaru et al. (2016)
		*P. mirabilis* (collection), *S. infantis* (collection)	
		*P. aeruginosa* ATCC 10145	
		*S. aureus* ATCC 6338	
Al_2_O_3_	11	*E. coli* MG 1655	Simon-Deckers et al. (2009)
Au	8.4	*A. baumannii*, *E*. *coli* J96, *E. coli* O157:H7, MRSA, *P. aeruginosa*, PDRAB, *S. aureus*	Lai et al. (2015)
		*E. faecalis*, *E. faecium*, *E. faecalis* VRE1	
		*E. faecium* VRE4	
	50, 100	*S. oneidensis* MR-1	Jahnke et al. (2016)
CeO_2_	6	*B. subtilis* ATCC 6333	Pelletier et al. (2010)
		*E. coli* ATCC 700926	
	15	*B. subtilis* ATCC 6333	
		*E. coli* ATCC 700926	
	22	*B. subtilis* ATCC 6333	
		*E. coli* ATCC 700926	
	40	*B. subtilis* ATCC 6333	
		*E. coli* ATCC 700926	
	7	*E. coli* RR1	Thill et al. (2006)
	2–4	*L. monocytogenes* ISP 6508	Tamayo et al. (2014)
Cu	20–100 nm	*S. aureus*	Kredl et al. (2016)
	6–9 nm	*B. subtilis*	
	25 nm	*B. subtilis*, *C. perfringens*, *P. aeruginosa*, *E. coli*, *S. aureus*, *L. monocytogenes, C. tropicalis*, *F. verticillioides*	
Cu_2_O	40	*E. coli*	Meghana et al. (2015)
CuO	22.4–94.8	*S. aureus* EMRSA-16, *S. aureus* (MRSA) 252	Ren et al. (2009)
		*S. aureus* EMRSA-15, *E. coli* NCTC 9001	
		*S. aureus* NCTC 6571	
		*S. aureus* ‘Golden’ (lab isolate), *S. epidermidis* SE-4 and SE-51	
		*P. aeruginosa* PAOI, *Proteus* spp. (lab isolate)	
	30	*E. coli*	Meghana et al. (2015)
MgO	4	*E. coli* C3000, *B. megaterium* ATCC 14581	Stoimenov et al. (2002)
		*B. subtilis* ATCC 6333	
	20	*E. coli* XL-1 blue	Leung et al. (2014)

### 7.3. Nano-drug delivery system in microbial biofilm control

Nanotechnology can be used in various ways to prevent, monitor, control, and cure diseases. These methods include combining materials or devices with drugs and biomolecules, adding advantages like slow and controlled drug release, promoting greater efficiency of tissue penetration, and encouraging greater protection against drug degradation (Lin et al., 2015). For the delivery of bioactive compounds, liposomes (LIPs), micro-emulsions (MEs), nanoemulsions, solid lipid nanoparticles (SLNs), polymeric nanoparticles (PNs), and metallic nanoparticles are currently the primary types of nanosystems used for the delivery of bioactive compounds (MNPs). The use of nanostructured systems for the treatment of infectious diseases that are persistent or resistant to traditional therapies has the potential to significantly increase patient quality of life and lifespan (Bharali et al., 2011).

It is becoming increasingly difficult to treat infections with traditional antimicrobials as microbes evolve resistance mechanisms and biofilms. Nanotechnology’s use of nano-carriers for the delivery of drugs and biomolecules for the prevention and treatment of bacterial biofilms is an encouraging technique for overcoming bacterial resistance (Pelgrift and Friedman, 2013; Varma et al., 2020). Nanotechnology-based drug delivery systems, on the other hand, can allow drugs to directly interact with the complex structure of biofilms and exert action at various points in the biofilm development process, expanding the systems’ potential utility in the treatment of biofilms.

Researchers primarily focus on two of these systems’ key capabilities: direct engagement with planktonic cells (single cells) and interaction with or denaturation of the EPS matrix. A drug delivery system using nanoparticles for drug release and their interaction during the biofilm formation stages is schematized in Figure 2C. Since direct engagement between nanoparticles and microbe membranes (e.g., lipid nanoparticles, LIPs, and others) improves drug access into intracellular medium, direct interaction of nanoparticles with individual cells may inhibit biofilm development. The last event (dispersion), when individual cells are released from the polymer matrix and are able to restart the biofilm formation cycle, can also be linked to this type of interaction. The nanoemulsions, LIPs, SLNs, lipoproteins, and micelles can have a direct impact on the biofilm polymer matrix, increasing nanoparticle fusion and triggering protein denaturation and lipid bilayer fusion. This makes it easier for the nanoparticles to enter the biofilm and facilitate contact with the microbial cells (Forier et al., 2014).

#### 7.3.1. Liposomes

Since delivering water-based drugs through biological membranes is thought to be challenging, a strategy has been devised to develop techniques of a similar type that can accomplish this task. To address this issue, some delivery technologies, like LIPs, were first examined (Al-Jamal and Kostarelos, 2011). The LIPs, which are utilized to transport desired medications to the body’s target areas, are small, spherical vesicles with a membrane made of a phospholipid bilayer or sphingolipids. The properties of these bilayer molecules, such as rigidity or fluidity, and the charge of the bilayer can be controlled depending on their composition. These bilayer molecules can be created from cholesterol (CHOL) or other benign phospholipids (Akbarzadeh et al., 2013).

Al-Jamal and Kostarelos (2011) assert that LIPs are well-established nanometric delivery systems for antifungals, cytotoxic medicines, vaccinations, and imaging agents. LIPs are more effective than other nanosystems because of their potential to load both lipophilic and hydrophilic medicines and their biodegradability, biocompatibility, lower toxicity, and lower dosages. On the other hand, in addition to their high prices, LIPs have some drawbacks, including low solubility, a brief half-life, and the potential for phospholipid oxidation and hydrolysis (Akbarzadeh et al., 2013).

Jones et al. (1997) studied cationic and anionic LIPs to transfer the hydrophobic bactericide triclosan (TCS) to bacterial biofilms. *Streptococcus oralis*, *Streptococcus sanguis* C104, *Streptococcus gordonii*, *Streptococcus salivarius* DBD, and *S. salivarius* 8,618 were tested against various bacteria using cationic LIPs with varying concentrations of dimyristoylphosphatidylcholine (DMPC), CHOL, and dimethyldioctadecylammonium bromide (DDAB). *Streptococcus salivarius* DBD demonstrated the highest level of cationic LIP absorption, while *S. sanguis* C104 demonstrated the lowest level of absorption. The best outcomes were seen against *S. sanguis* C104 for the anionic LIPs, which were made of DMPC and phosphatidylinositol (PI). However, the tactic failed against *S. salivarius* biofilm. Using LIP formulations greatly affected the different species in the biofilms, showing how crucial electrostatic contact is for TCS delivery. Catuogno and Jones (2003) investigated the antibacterial activity of zinc citrate particle-produced solid-supported LIPs against *S. oralis* biofilms, the most prevalent oral bacterium. Meropenem (MER) was enclosed in anionic and cationic LIPs, and (Drulis-Kawa et al., 2009) investigated the variations in how they interacted with *P. aeruginosa* biofilms. The findings showed a strong interaction between nanoparticles and bacterial cells, particularly cationic LIPs. This is consistent with the positively charged LIPs’ interaction with the negatively charged exterior membrane of microbes. Also, the hydrophobic parts of the membrane might help the LIPs connect better to the membranes of bacterial cells. Gubernator et al. (2007) investigated the *in vitro* antibacterial activity of cationic LIPs that contained the antibiotics ciprofloxacin (cipro), meropenem (MER), or gentamicin (GEN) against the clinical strains of *P. aeruginosa, Klebsiella pneumoniae*, and *E. coli*. When Cipro and MER were administered using LIPs, encouraging outcomes were seen. *Pseudomonas aeruginosa* and a number of other Gram-negative bacteria, including *Bordetella bronchiseptica*, *E. coli*, *K. pneumoniae*, *Acinetobacter lwoffii*, and *Acinetobacter baumannii*, were the focus of research conducted by Alipour et al. (2008) who investigated the effect of liposomal POLY B. According to the research group’s findings, all of the microorganisms displayed a better sensitivity profile when the drug was employed in combination with LIPs than when the free drug. This was especially true for the resistant strain of *P. aeruginosa* (PAM13641-1).

The liposomes are made of one or more phospholipid bilayers to prevent reticuloendothelial rejection and penetrate biofilms. The phospholipid bilayer has a hydrophilic head for liposome surface characteristics and a hydrophobic tail for fluidity (Wang et al., 2020). Hydrophobic (lipophilic) antimicrobials can be put into phospholipid bilayers, while hydrophilic drugs can reside in the aqueous core. Encapsulating antimicrobials in the aqueous core of liposomes shields them from deactivation agents *in vivo* with high drug delivery by lipid bilayer fusion with bacterial cell membranes, releasing the antimicrobial directly into bacteria (Baek et al., 2018). Their effectiveness against biofilms depends mainly on their size and charges. Cationic, unilamellar, and smaller liposomes can disrupt the biofilm’s electrostatic balance and get inside it (Dong et al., 2015). Liposomes have outstanding biocompatibility, low toxicity, may carry both hydrophobic and hydrophilic medicines, and are biodegradable. However, their packaging stability, short half-life, low solubility, poor drug loading efficiency, the potential for phospholipid oxidation and hydrolysis, high cost of manufacture, and low drug loading efficiency limit their production (Baek et al., 2018).

#### 7.3.2. Microemulsions and nanoemulsions

Microemulsions (MEs) as a method of drug delivery have gained prominence in the field of pharmaceutical research due to their ability to effectively distribute a large range of molecules with varying characteristics to different parts of the body. The MEs are transparent emulsions with water or oil microdroplets in water microdroplets. Surrounding nanometric droplets in an internal phase with a surfactant or amphiphile, often combined with a co-surfactant, creates a thermodynamically stable system (Bonifácio et al., 2014). MEs are reservoirs that release the active component after isolating it from the dissolving media. The capacity of MEs to detach from a constrained environment and form bonds with various chemical substances is one of their distinguishing characteristics. Their adaptability, such as low surface tension, enhances the solubility, stability, and bioavailability profiles of the linked molecules, leading to a rise in their absorption and penetration. It becomes necessary to add a co-surfactant when a surfactant cannot produce nanometric droplets to increase the surface area along with reduce the particle size and improves the therapeutic impact (Lawrence and Rees, 2000).

MEs are beneficial because they can be formed spontaneously and have outstanding thermodynamic stability. It also has a good look and a high drug-loading capacity, which improves bioavailability and reduces toxicity. Moreover, microbes cannot exist in pure fat or oil; hence these MEs systems are also antibacterial. Some studies demonstrate that MEs’ structure contributes in antibacterial activity by focusing on the bacterial cytoplasmic membranes (Al-Adham et al., 2013). Furthermore, MEs are the prime choice for nanotechnology-based systems for numerous reasons. First, thermodynamically stable systems can spontaneously develop without energy. They can load hydrophilic and lipophilic medicines, boosting efficiency and decreasing dose and adverse effects. MEs cannot solubilize high-melting compounds. When creating droplets, a large quantity of surfactants is required to keep them stable, and their stability relies on pH and temperature (Lawrence and Rees, 2000).

**Figure 5:**
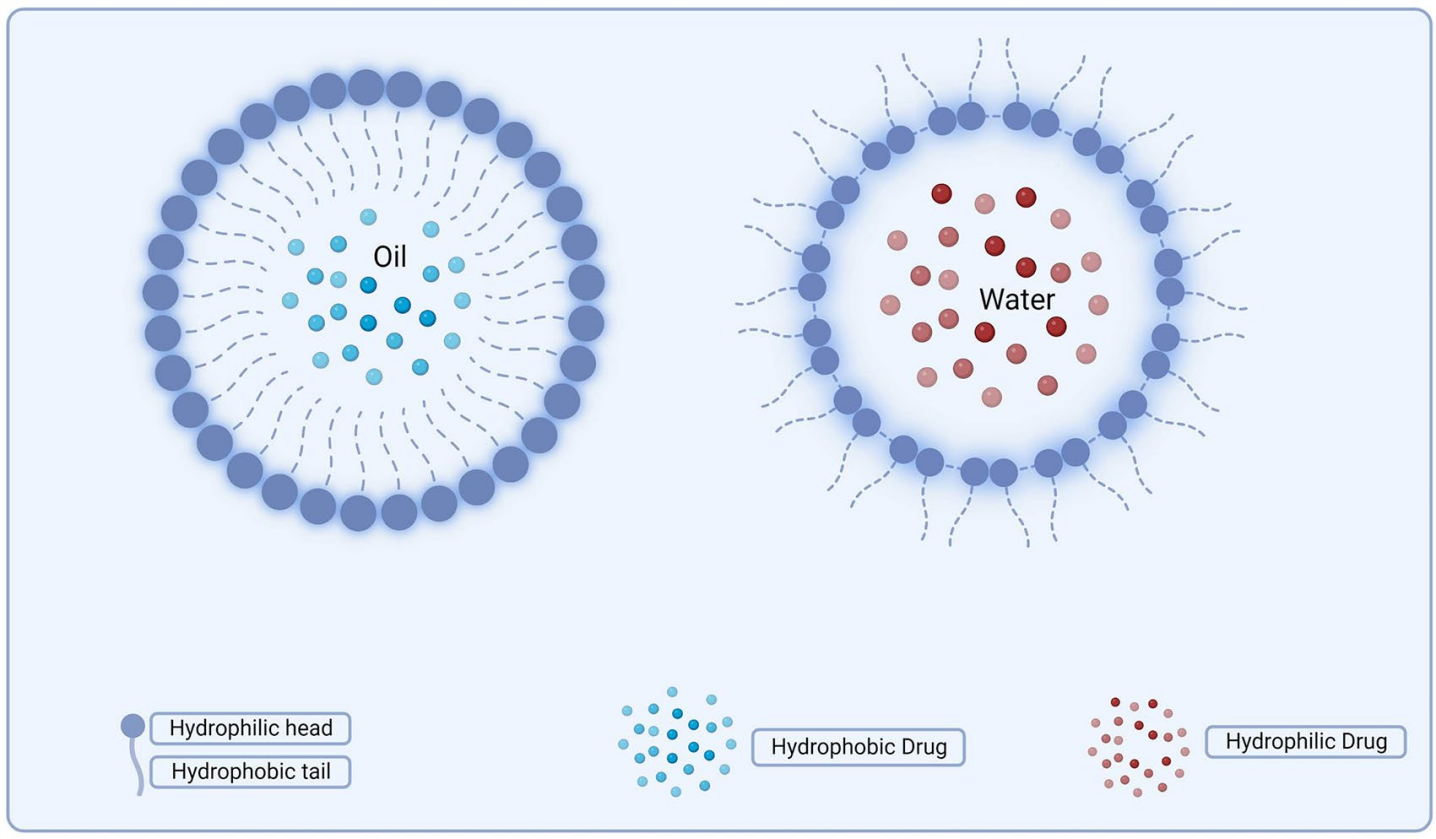
Structures of microemulsions and nanoemulsions.

Nanoemulsions (NEs), like MEs, are heterogeneous systems in which the inner phase liquid is dispersed as droplets in the outer phase. Nanoemulsions’ physicochemical properties are impacted by their qualitative and quantitative components (Hung et al., 2007). Literature is inconsistent about nanoemulsions and MEs. Both systems are comparable structurally and visually yet thermodynamically unstable. MEs are more stable than nanoemulsions. Ironically, MEs have smaller droplets than nanoemulsions (despite their name). Nanoemulsions may be used as drug delivery methods for hydrophobic compounds having low water solubility despite their low thermodynamic stability (Hung et al., 2007). The structure of the microemulsion and nanoemulsions are depicted in Figure 5.

Various research groups have carried out several studies on MEs and nanoemulsions. Ramalingam et al. (2013) analyzed the effects of a cetylpyridinium chloride (CPC)-containing oil-in-water nanoemulsion on the biofilms that form on the waterlines of dental units. Using the microdilution technique and the biofilm assay, the activity of CPC loaded nanoemulsion was tested against planktonic *C. albicans*, *S. mutans*, *Lactobacillus casei*, and *Actinomyces viscosus*. Moreover, the nanoemulsion was also tested against a mixed culture. The results revealed that when the formulation was applied to microorganisms, both singly and in a mixed culture, the activity increased. This was compared to the components alone, which exhibited a similar activity level. The efficacy of MEs and nanoemulsions to suppress the production of biofilms by *S. aureus* NCTC 1803, *S. typhimurium* PSB 367, *Listeria monocytogenes*, *P. aeruginosa*, and *E. coli* O157:H7 was also investigated (Teixeira et al., 2007). MEs were also highly active against *P. aeruginosa*, a noso-comial bacteria with a high level of resistance to antibiotics. Although both systems were able to remove biofilms produced by pathogenic bacteria mentioned above, MEs were the only system that could remove biofilms produced by *P. aeruginosa*. On the other hand, it was discovered that *L. monocytogenes* are resistant to both mechanisms.

Curcumin (CUR)-loaded myristic acid-based MEs were created by Liu and Huang (2012) to explore the antibacterial efficacy of these compounds against skin infections induced by *S. epidermidis* BCRC 11030. The myristic acid may be useful as a vehicle for the ME to load CUR since it exerted a synergistic inhibitory impact on *S. epidermidis* biofilms when CUR was loaded into the ME combined with it. Therefore, it appears that myristic acid may serve as a useful medium for loading CUR into the MEs. It has been demonstrated in some research that MEs composed exclusively of antimicrobial ingredients are more effective at dissolving microbial biofilms (Al-Adham et al., 2003).

#### 7.3.3. Solid lipid nanoparticles

Colloidal drug-delivery systems known as solid lipid nanoparticles (SLN) are made of solid lipids (one or more) which are stabilized by surfactants. Although SLNs are almost identical to nanoemulsions having very low cytotoxicity that include a solid lipid core at room temperature, which gives the drug less mobility and allows for greater control of the release of the drug. The size control, increased stability, the freeze-dry, and refurbish ability, high efficiency of drug loading, and the most importantly inexpensive manufacturing, are all the prime features of SLNs (Pircalabioru and Chifiriuc, 2020). As, SLNs can efficiently invade biofilms and mucus layers, it is preferable to use as delivery vehicles for medications that cannot pass through these barriers. The SLNs have anti-virulence qualities (Nafee et al., 2014); but there may be some restrictions on SLN systems that reduce their effectiveness. For instance, the lipid shell may affect how an antibiotic binds to the bacterial outer membrane, reducing its antibacterial effectiveness compared to an antibiotic without encapsulation (Li et al., 2019a). The drug’s loading is also negatively impacted by its solubility in the solid lipid (Al-Wrafy et al., 2022).

**Figure 6:**
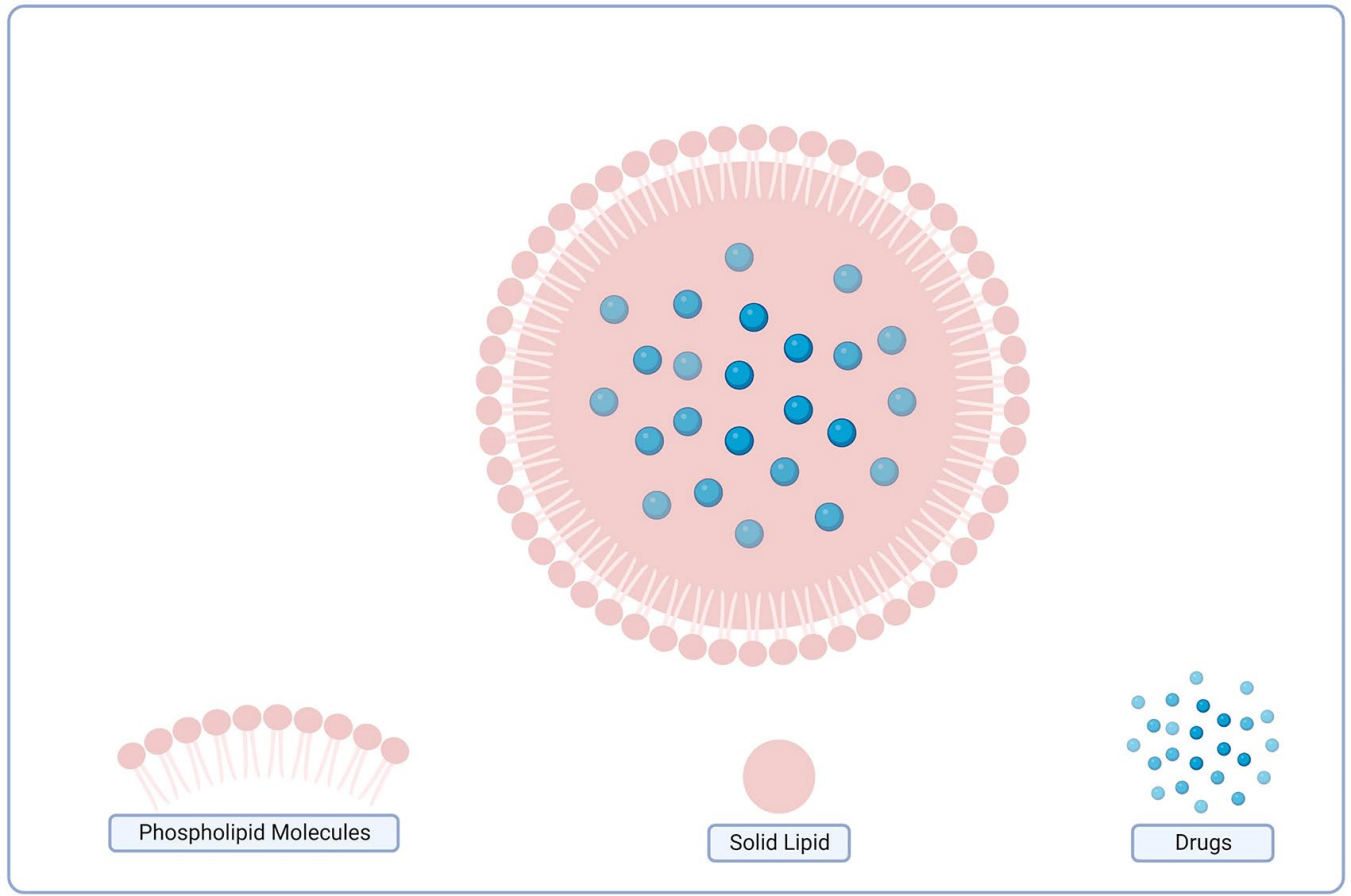
Structure of the solid lipid nanoparticles.

SLNs display the characteristics of various colloidal carriers. The sustained release of pharmaceuticals from lipid matrix, such as polymeric nanoparticles, can be achieved with the help of physiologically acceptable substances like emulsions and LIPs. The scale-up and manufacturing of SLNs are highly attractive due to (i) integrating lipophilic and hydrophilic drugs, (ii) physical stability, (iii) sustained drug release, (iv) cytocompatibility, (v) site-specific drug delivery, (vi) enhanced drug stability, (vii) improved formulation stability, (viii) freeze-dry and reconstitute ability, (ix) a high drug payload, (x) adjustable particle size, (xi) preventing carrier toxicity, and (xii) low cost of production (Dos et al., 2018). The structure of the solid-lipid nanoparticle is illustrated in Figure 6.

The SLN approach, however, also comes with some drawbacks. These include drug load restriction due to solid lipid solubility, drug expulsion when lipid crystallizes, and particle concentration in aqueous dispersions varying from 1% to 30% (Dos et al., 2018).

SLNs are made of SA, lauric acid (LA), and oleic acid to prevent and treat nosocomial infections in surgical implants (OA). The plan was to use SLNs to weaken bacterial adhesion to surfaces and tissues, reducing the development of biofilms. Additionally, LA and OA’s antimicrobial effects induced bacterial cell membrane breakdown. Scientists argue surface roughness, reduced contact area, and weakened bacterial attachment mechanism lowered adhesion. Furthermore, few bacteria that adhered had destabilized walls due to SLN’s antibacterial fatty acids. Due to the multidrug resistance of bacteria to antibiotics, it is hypothesized that it is most important to look for novel options, like SLNs, that are independent of the activity of antimicrobial drugs. These SLNs can be put on surgical implants as coatings to stop biofilms from growing (Taylor et al., 2014).

To maximize the delivery of QSIs in the lung tissue of patients with *CF*, (Nafee et al., 2014) created SLNs through hot-melt homogenization with glyceryl palmitostearate, glyceryl behenate, and TRI. Since QSI is lipophilic, its integration into SLNs was made easier, and its encapsulation efficiency ranged from 68% to 95%. With the aid of simulated lung fluid and phosphate-buffered saline (PBS) buffer, the release of QSI from various SLNs was investigated (SLF). With 60–95% of the drug released over 8 h, a regulated release of QSI in PBS buffer was seen. The release of QSI was sustained in SLF, displaying a >20% burst in all SLNs, making it a more pertinent and superior medium for *in vivo* settings. According to the authors, SLN therapy inhibited *P. aeruginosa*’s proliferation. The authors claim that SLNs that were effectively loaded with the novel QSIs could be created with a payload release that lasted longer than 8 h. Because of the hydrophilic surface of SLNs, QSIs could readily penetrate the mucus and serve as drug carriers for *CF* therapies. As a result, the application of SLNs represents a fresh viewpoint in the nano-based delivery of innovative anti-infectives.

#### 7.3.4. Polymeric nanoparticles

**Figure 7:**
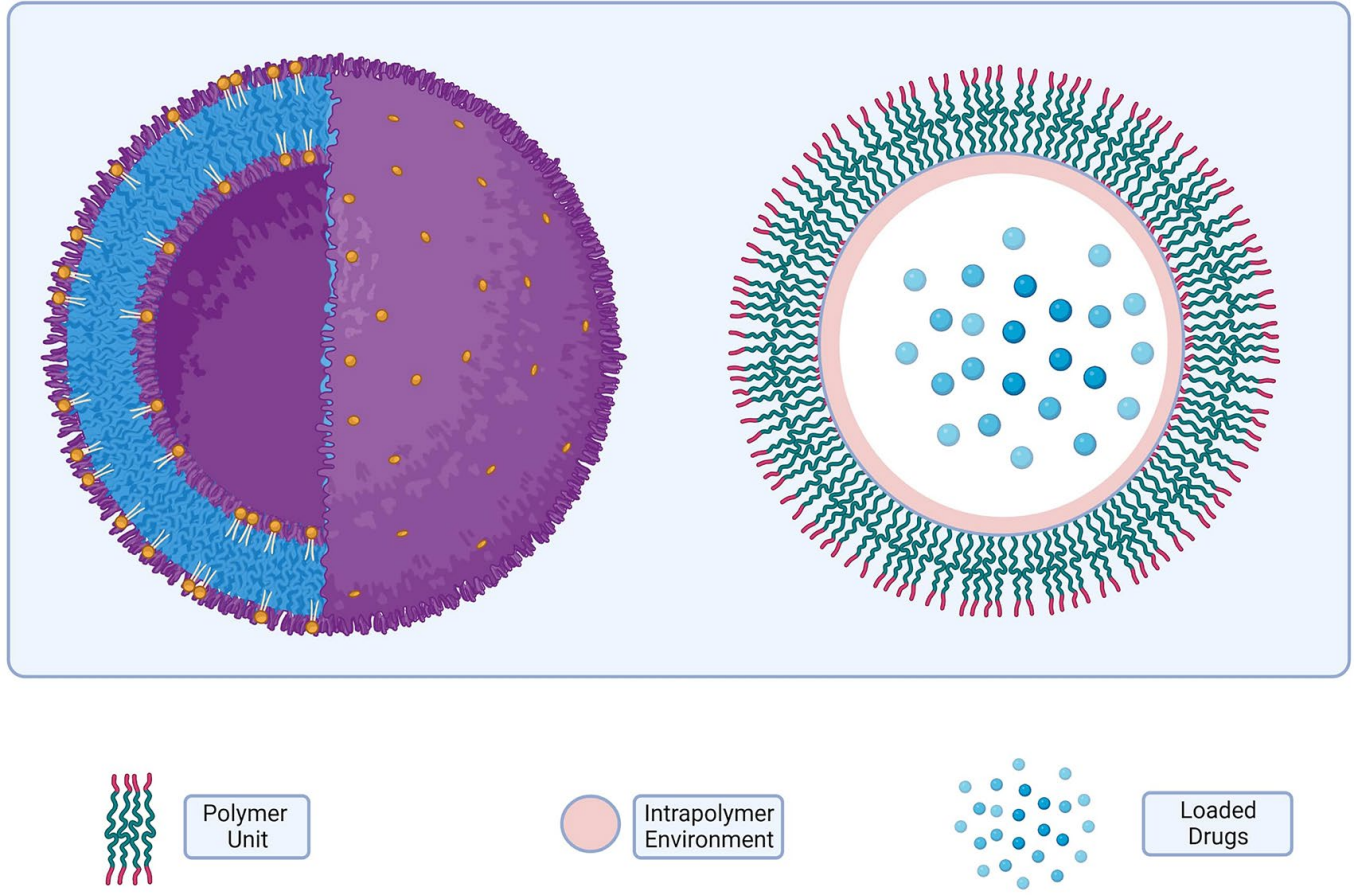
Structure of polymeric nanoparticles used in active drug delivery to the target cells.

Several research organizations have also studied polymeric nanostructures (Figure 7) with intrinsic anti-biofilm characteristics. EPS in bacteria and biofilms have a negative charge on its outer layer, and this substance works through electrostatic interactions (Huang et al., 2011; Renner and Weibel, 2011). The charged polymer chitosan is widely employed because of its excellent antibacterial action. For the root canal, chitosan nanoparticles were produced and evaluated, which prevented biofilm production in single and mixed-species biofilms, respectively, by 97% and 94%. For an additional 8 days (5-log decrease), the chitosan nanoparticles progressively destroyed a pre-formed mixed-species biofilm (Elshinawy et al., 2018). A 7-day-old *E. faecalis* biofilm was investigated using chitosan nanoparticles in another investigation. Using chitosan nanoparticles at a concentration of 5 mg/mL for 72 h of incubation at 37°C, the authors discovered that 4 logs reduced bacteria’s viability, and at a dosage of 20 mg/mL, the majority of the bacteria were killed (Shrestha et al., 2010). Cationic PLGA nano-polymer inhibited the development of Streptococcus mutants for 24 h, with most bacteria dying within 90 min of exposure. At a 100 g/mL concentration, the cationic nano-polymer killed 73 percent of the bacteria and completely destroyed 1-day-old biofilms (Zhu et al., 2018). Harper et al. (2019) coupled the effects of electrolyte charge screening with anionic (+) alpha-tocopherol phosphate (-TP) liposome nanoparticles in different research to improve the diffusion of the latter through a biofilm. Prior to treatment with nanoparticles, the bacterial biofilm had formed for 18 h. A phosphate (−ve) buffer was used to study the ability of 700 nm self-assembled liposomes to penetrate the multispecies oral biofilms from a donor. Using a tris (hydroxymethyl) aminomethane (+ve) buffer, the liposomes could penetrate the biofilms and destroy the bacteria trapped within.”

To test the nanoparticles’ *in vitro* and *in vivo* efficacy against *P. aeruginosa* infection, a method was devised to enhance GEN retention in PLGA nanoparticles (Abdelghany et al., 2012). The authors believe that both nanoparticles and microparticles could be useful in aerosol delivery. In treating lung infections, they may improve the effectiveness of GEN and other aminoglycosides. This potential application of nanoparticles and microparticles in aerosol delivery was found to enhance the therapeutic effectiveness of GEN and other aminoglycosides.

To improve antibacterial activity against planktonic and biofilm forming *H. pylori*, Cai et al. (2015) created lipid polymer nanoparticles (LPNs) to carry amoxicillin drug. The outer lipid bilayer of the PNs, can readily integrate into the polysaccharide matrix of the biofilm, and subsequently remove this protective layer of the bacterial milieu, allowing amoxicillin to directly go into the free bacterial cells. Pectin sulfate (also known as PEC) and a mixture of lipids (i.e., rhamnolipids and phospholipids), make up the bulk of the PNs. Antibacterial activity was enhanced by the presence of rhamnolipids and phospholipids, which, due to their anti-adhesive properties, made biofilm formation tough. The PEC suppressed the growth of *H. pylori* by vying with specific target cells. According to the results of fluorescence tests, bacteria that were treated with LPNs were unable to invade AGS cells. In contrast, ~100% of AGS cells were infested and lysed when free amoxicillin was present. This indicates that LPNs could be an effective method for tissue defense against *H. pylori* and a tool for preventing the development of bacterial biofilms.

Takahashi et al. (2015) designed and examined the antibacterial potential of PLGA-PNs and chitosan-associated PLGA (CS-PLGA)-PNs containing CAM in *S. epidermidis* biofilms. During the study, the PLGA-PNs containing CAM (62%) demonstrated the highest antimicrobial potential. Images of biofilms treated with PLGA-PNs having CAM, on the other hand, showed a significantly degraded and disrupted matrix with numerous pores. Furthermore, a decrease in biofilm thickness was detected, indicating bacterial mortality. The authors concluded that PLGA-based PNs could be excellent CAM carriers in anti-biofilm treatment.

### 7.4. Carbon-based nanomaterials

Carbon nanodots are a novel form of NM that emerged in the recent decade (CNDs). Their one-of-a-kind surface chemistry, diminutive size, and photoluminescent properties made them useful in many contexts (Chatzimitakos et al., 2017; Chatzimarkou et al., 2018). CNDs have been singled out for potential bioimaging uses in the medical field because of their great biocompatibility. Despite appearances, not all substances that are harmless to eukaryotic cells are also harmless to prokaryotic cells. In order to take advantage of this idea, recent research has focused on the antibacterial capabilities of CNDs. CNDs have carbon and oxygen groups in their simplest form, yet depending on the precursor materials, heteroatom doping may occur. CNDs’ ability to inhibit bacterial growth stems from the wide variety of functional groups present in the compounds. Vitamin C was used as a precursor in the electrochemical synthesis of CNDs by Li et al. (2018). Staphylococcus aureus (*S. aureus*), Bacillus subtilis (*B. subtilis*), Bacillus sp. WL-6, and Escherichia coli (*E. coli*) were the gram-positive and gram-negative bacteria tested, respectively (*E. coli*). Results showed that a concentration of 50 g/mL was sufficient to completely suppress colony formation for the *Bacillus* species (i.e., *B. subtilis* and *Bacillus* sp. WL-6), whereas for the other two species, a concentration two times higher was required. CND treatment of ampicillin-resistant *E. coli* had the similar outcomes. These findings show the vitamin C CNDs’ broad-spectrum antimicrobial action. The authors aimed to figure out how the bacteriostatic effect is created. CNDs were discovered to be taken up by bacteria *via* diffusion, but they also coated the bacterium’s membrane, making it rougher and leading in the bacteria being cut off from the growth media and the leaking of intracellular components. Internalized CNDs were discovered to connect to DNA *via* noncovalent bonds, which resulted in the DNA’s loosening and unwinding (alteration of the secondary conformation). RNA showed identical behavior. Compared to antibiotics, the benefits of NMs and CNDs are especially clear when considering their antibacterial action, which is mediated by a multifaceted mechanism. Finally, another benefit of vitamin C-derived CNDs is that they decompose quickly into CO_2_, CO, and H_2_O when exposed to visible light, mild temperature (37°C), and air. Microorganisms have the capacity to undergo this breakdown on the inside. Therefore, CNDs are destroyed and can no longer constitute a hazard to other microorganisms after killing or limiting bacterial growth. The DNA binding characteristics of the CNDs from tamarind were observed to suppress the growth of *Klebsiella pneumoniea*, *Escherichia coli*, *Staphylococcus aureus*, and *Pseudomonas aeruginosa* (Jhonsi et al., 2018). To replicate this, Jian et al. (2017) used a pyrolysis approach to manufacture CNDs from three biogenic polyamines (putrescine, spermidine, and spermine). The biogenic polyamines were chosen because of their ability to reduce the MICs of-lactams for many different bacteria, but their limited use as surface modification agents. The authors tested the efficacy of spermidine-derived CNDs and pure spermidine against methicillin-resistant (MRSA). It was shown that the MIC for *Staphylococcus aureus, enterococci, Pseudomonas aeruginosa,* and *Escherichia coli* CNDs formed from spermidine was 2,500 times lower than that for pure spermidine. Binding with membrane components (such phospholipids, porins, peptidoglycans, etc.) was the primary mechanism by which the antibacterial action was produced. It was hypothesized that the “ultrahigh” positive charge of the CNDs was responsible for the strong binding affinity. CNDs generated from spermidine have the potential to bind to DNA and siRNA, inhibiting vital bacterial functions including DNA replication and gene expression and so reducing the bacteria’s chances of survival. Authors used spermidine-derived CNDs to treat bacterial keratitis in infected rabbits (Jian et al., 2017). Paracellular transport of CNDs was facilitated by the opening of tight junctions in corneal epithelial cells, providing further proof of their suitability for treating bacterial keratitis. This research not only proves the usefulness of CNDs as antibacterial agents, but also reveals how simple chemical compounds may be modified to yield CNDs with novel capabilities. Aminoguanidine and citric acid CNDs showed similar findings, in that they could limit the formation of both the biofilm and the planktonic *P. aeruginosa* (Otis et al., 2019). The interactions between aminoguanidine residues on the surface of CNDs and the lipopolysaccharides of *P. aeruginosa* are thought to be responsible for their selectivity toward *P. aeruginosa* over *S. aureus*, *B. cereus*, *E. coli* K12, *E. coli* DIHO B, *Salmonella typhimurium* strain ATCC14028. Microwave treatment of graphene oxide for 9 h at 650 watts in the presence of concentrated acids (HNO_3_ and H_2_SO_4_) yielded CNDs (Sun et al., 2014), which exhibited antibacterial activity *via* a mechanism distinct from that of graphene oxide. They were able to kill off *E. coli* and *S. aureus* because of their peroxidase-like activity, which caused hydrogen peroxide to split into hydroxyl radicals. This is proven by the fact that the antibacterial activity attained in the combination treatment with hydrogen peroxide and CNDs is higher than that achieved with hydrogen peroxide alone. This is due to the fact that hydroxyl radicals are far more effective in killing bacteria than hydrogen peroxide. Advantages of this method include both improved wound disinfection and reduced risk of toxicity from hydrogen peroxide at high concentrations, which is currently the standard method of wound disinfection. As this research shows, CNDs may be used to boost the antibacterial activity of well-known chemicals in a roundabout method, allowing for new uses.

### 7.5. Metallic organic frameworks

Given its high porosity, huge surface area, and photocatalytic feature, MOFs can also be employed for air filtering. Traditional MOF air filters operate as physical barriers to remove particulate matter and bacteria/fungi from the air, but they cannot kill or eradicate the microorganisms. Li et al. studied a series of MOFs, including MIL-100 (Fe), NH2-MIL-125 (Ti), NH2-UIO-66 (Zr), zeolitic imidazolate framework-11 (ZIF-11) (Zn), and ZIF-8 (Zn), and demonstrated that ZIF-8 enabled almost complete inactivation of *E. coli* with over 99.9999% inactivation efficiency in saline with 2 h solar irradi Inhibiting *E. coli* growth was predicated on the fact that ROS were produced when photoelectrons were trapped at the Zn + centers of ZIF-8 *via* the ligand to metal transfer. With a photocatalytic killing effectiveness of >99.99% against airborne germs in 30 min and 97% particulate matter removal, the ZIF-8 air filter demonstrated exceptional air cleaning performance (Li et al., 2019b). It was discovered that this MOF-based filter is quite efficient since it can filter out infections and other contaminants at the same time (e.g., particulate matter and bioaerosols). Therapeutic effects against bacteria have been enhanced by incorporating MOFs with antibacterial drugs and NPs. AgNPs or Ag^2+^ ions are a common example due to their well-known antibacterial capabilities. Despite the fact that AgNPs have been extensively investigated for antibacterial applications, there are a number of limitations connected with their production and usage, including the lack of controllability of size and shape, aggregation, colloidal stability, and possible toxicity (Zhang et al., 2019). MOFs, with their homogeneous and porous architectures, are good templates for the inclusion of AgNPs, which can help relieve these issues. For instance, Shakya et al. (2019) recently produced a γ-cyclodextrin metal–organic framework (MOF) embedded with AgNPs (Ag@CD-MOF) by a reaction–diffusion approach and subsequently functionalized with the fibrinogen-mimetic peptide, Gly-Arg-Gly-Asp-Ser (GRGDS) to generate GS5-CL-Ag@CD-MOF for combination anti In comparison to testing with non-functionalized Ag@CD-MOF, the composite material exhibited significantly improved hemostatic activity, decreasing blood clotting time by 39.5%. In contrast to the control Ag@CD-MOF, which took almost 2 weeks to have any noticeable impact, the GS5-CL-Ag@CD-MOF composite resulted to a 90% reduction after wound size in 10 days (Shakya et al., 2019). Photodynamic antibacterial treatment using metal–organic frameworks (MOFs) has seen widespread use of photosensitizing porphyrins (Zhou et al., 2018). Porphyrynic (PCN-224) MOFs were loaded with Ag ions, and then coated with hyaluronic acid (HA) to create a synergistic photodynamic and on demand antibacterial action against gram-positive bacteria. In response to contact with MRSA bacteria exuding hyaluronidase (HAase), the HA on the nanocomposite’s surface degraded, exposing the positively charged PCN-224-Ag^2+^ and allowing it to efficiently attach to germs. In addition, visible light irradiation of the MOF nanostructure resulted in the production of ROS, which in turn killed off the bacteria (Zhang et al., 2019).

MOFs may also have an antibacterial photodynamic treatment (PDT) impact, complementing the use of chemotherapy to eradicate germs. In addition to its traditional uses in the treatment of diseases like skin diseases and cancer, PDT has recently seen increased usage in the field of antibacterial applications (Yan et al., 2017; Li et al., 2020). PDT’s benefits include being non-invasive, having few to no negative side effects, and having a low propensity to generate bacterial resistance. Light irradiation at specific wavelengths activates reactive oxygen species (ROS), which can severely harm tumor cells and dangerous microorganisms (Teng et al., 2021; Zhou et al., 2021). It was demonstrated that the ROS produced might cause significant harm to bacteria and significantly slow their development. To do this, Chen M. et al. (2020) synthesized a PCN-224 MOF out of ZrCl4 and tetrakis (4-carboxyphenyl porphyrin), then included Ti to partially substitute the Zr clusters (Zr/Ti) to generate PCN-224 (Zr/Ti) with a size of around 400 nm. This bimetallic MOF shown enhanced photocatalytic activity, leading to strong reactive oxygen species (ROS) production and outstanding antibacterial efficiency against bacteria, even those resistant to several antibiotics. It was hypothesized in this work that the strong antibacterial performance was due to the rupture of the outer membrane, and this hypothesis was supported by the results of the early mechanism exploration.

As shown in their research, Au-Duong and Lee have also loaded naturally antibacterial chemicals like iodine into MOFs. ZIF-8 was employed for this study. *Escherichia coli, Staphylococcus epidermidis*, and *S. aureus* were used in experiments with the hybrid material. The results showed that the antibacterial impact was pH-dependent, with iodine being most efficiently liberated from the ZIF-8@I nanocomposite during its acidic breakdown at a pH of 6 (Au-Duong and Lee, 2017). Wei et al. also developed a sodium-doped mesoporous MOF-Prussian blue of 200 nm size for the treatment of deep bacterial osteomyelitis (Wei et al., 2021). Prussian blue, which responds well to microwaves, has been discovered to have a high heat-to-energy conversion efficiency. Additionally, ROS can be produced *via* Fenton reactions and glutathione consumption inside bacteria, leading to bacterial mortality. The copper MOF-CuBSC was the primary structure of a biomimetic structure created by Cheng et al., which encapsulated glucose oxidase and L-arginine. Glucose oxides promoted the oxidation of glucose, producing H_2_O_2_, oxygen radicals, and NO gas, and the resulting hybrid structure showed remarkable efficacy as an antibacterial agent against both Gram-negative *E. coli* and Gram-positive *S. aureus* (Cheng et al., 2020).

## 8. Limitations of nanomaterials in biological applications

Nanoparticles are intriguing and could be useful from an engineering or biological point of view since they have qualities that are one of a kind, such as a tiny ratio of surface area to volume. In a similar vein, the features that might result in unanticipated toxicities are noteworthy in their own right. The toxicity level of anionic nanoparticles is significantly lower than that of cationic nanoparticles, which include gold and polystyrene nanoparticles (De Jong et al., 2008). It has been shown that cationic nanoparticles can induce hemolysis and coagulation. Nanomaterials have several entry points into the body, including the skin, the respiratory tract, parenteral administration, and others. When it enters the bloodstream, it will interact with the plasma proteins there, which will very certainly result in the creation of a protein corona. This protein corona has the potential to change the pharmacological characteristics of the nanoparticles. Due to the fact that toxicity is a primary issue (Bencsik et al., 2018), it is important to conduct an accurate analysis of the interaction that occurs between the nanoparticle and the body. Studies of nanoparticles conducted *in vivo* and *in vitro* have demonstrated that the modest toxicities seen are attributable to increased ROS levels and disrupting the host’s homeostasis (Khanna et al., 2015). The ROS might cause even more damage to the DNA and set up circumstances of oxidative stress, which would then lead to the production of micronuclei. Regardless of the size of the silver nanoparticles and quantum dots, it is likely that macrophages will be able to consume them, which will result in an increase in the release of inflammatory mediators such as TNF-α, MIP-2, and IL-1 (Albanese et al., 2012). Because nanoparticles have a propensity to collect in the liver, it is important to explore the specific mechanisms that govern how these particles are expelled from the body (Parveen et al., 2012). Platelet aggregation can be induced by both single-walled and multi-walled carbon nanotubes, but it cannot be induced by the C-60 fullerenes that make them up. In order to ensure efficient and reliable delivery of drugs, the underlying idea of how nanomaterials work will be discussed in depth. Nanoparticles are finding more and more applications as time goes on. Concerns of toxicity must also be taken into consideration.

## 9. Challenges and future prospects

The hunt for solutions for treating and controlling biofilm-related microbial illnesses is challenging. The use of NPs provides a suitable solution to this daunting hazard. However, the poor colloidal stability of NPs can limit their applicability, coupled with the fact that many such particles tend to form aggregates in solution and precipitate out. In addition, there are several aspects that serve as major drawbacks in the application of nanotechnology for combating biofilms. According to the most recent research, very little information is available about the effect that NPs have on the human body, which may serve as a major hindrance in utilizing nanoparticles for drug delivery. The environmental persistence of nanomaterials and their short, medium, and long-term implications and impacts remain unknown (Khan et al., 2021). It is crucial to conduct in-depth studies of NPs over extended periods to learn more about their mode of action and how they affect bacterial cells and biofilms. To treat oral biofilm infections, topical antimicrobials must persist on tooth surfaces for a long time and eradicate the biofilm microenvironment. Getting a firm grasp on how long NPs stick around during therapy is crucial. The ability of bacteria to withstand NPs is an important topic for further study. In addition, the ecological and genetic characteristics of biofilm producing microorganisms need to analyzed to detect resistant strains; this can help design standard protocols to fight such organisms. Consumer acceptance is also a critical point of analysis in the risk assessment of nanoparticles in medical implants as well as in the food industry (Martínez-Suárez et al., 2016; Gkana et al., 2017).

## 10. Conclusion

It is challenging to find effective treatments and management strategies for microbial illnesses caused by biofilms. Drug delivery *via* nanotechnology has the potential to be employed in the future to treat microbial biofilms. Fascinatingly, nanoparticles have the potential to synergize active compounds for biofilm inhibition, allowing for more efficient use of therapeutically available medicines by resolving issues with bioavailability.

The cost burden of treating infections caused by biofilms is substantial, and its incidence, severity, and morbidity have all been on the rise. Because of the difficulty in diagnosing, tracking, and evaluating the efficacy of novel medications for treating biofilm infections, more study is required. Cleaning medical equipment with nanoparticles is a viable option, and they can also be utilized to mitigate the spread of biofilms and prevent their formation. These NPs are harmless to biofilm and human cells, and they selectively target bacteria. Because of our newfound knowledge of the role of biofilm in the development of antibiotic resistance, we can employ NPs to boost medication delivery to cells. Adding specific charges and functional groups to NPs allows them to be guided toward certain biofilm components, where they can disperse the biofilm. Low colloidal stability may be to blame for NPs’ limited utility. Metal oxide and a biocompatible polymer are needed to stabilize NPs in suspension, which increases their anti-biofilm activity (serving as a stabilizer). Nanoparticles are being developed for a wide variety of novel applications, such as the removal of biofilms and the disruption of the quorum sensing mechanism. Extensive studies are being conducted to better understand the absorbance, metabolism, and degradation of nanoparticles, which are currently regarded as major knowledge gaps. These considerations point to NP’s possible future usefulness, albeit more study, particularly in the form of clinical trials, is needed to properly comprehend its spectrum of application.

Finding effective ways to treat and manage microbial diseases caused by biofilms is difficult. As nanotechnology advances in drug delivery, it may 1 day be used to combat microbial biofilms. The potential for nanoparticles to synergize active chemicals for biofilm inhibition is fascinating since it allows for more effective use of clinically available treatments by addressing bioavailability problems.

There has been a rise in the prevalence, severity, and morbidity associated with infections caused by biofilms with significant financial burden associated with biofilm infections in healthcare systems. Due to the complexity of these diseases, additional research is needed to determine how to diagnose biofilm infections, monitor their development, and assess the efficacy of new treatment drugs over time. Nanoparticles can be used to clean medical equipment, slow the growth of biofilms, and prevent new biofilms from forming. These NPs are safe to use, as they are biocompatible, selective for bacterial cells, and non-toxic biofilm. Now that we understand how biofilm contributes to antibiotic resistance, we can use NPs to improve drug delivery to cells. Through the incorporation of charges and functional specific groups into NPs, it is possible to direct the NPs to interact with targeted components of biofilm, leading to their dispersal. The limited usefulness of NPs may be due to their low colloidal stability. Stabilizing NPs in suspension for enhanced anti-biofilm activity requires both metal oxide and a biocompatible polymer (serving as a stabilizer). Nanoparticles are being produced for new and exciting uses, including the elimination of biofilms and the disruption of the quorum sensing mechanism. Extensive research is being done to fill the knowledge gaps that still exist about the absorbance, metabolism, and breakdown of nanoparticles. These factors highlight the potential relevance of NP in the future, although additional research, especially in the form of clinical trials, is necessary to fully understand its scope of applicability.

## Author contributions

YM, IC, and AM: conceptualization, methodology, investigation and manuscript draft. SM: prepared the images. HC, RA, BM, KP, and SKA: writing–review and editing. MS, TM, and NS: writing–review and editing. All authors contributed to the article and approved the submitted version.

## Conflict of interest

Author IC is employed by Indegene Pvt. Ltd. recently; however, during the manuscript preparation/initial submission, she was employed at USTM and declare that the work was without any commercial or financial relationship that could be a potential conflict of interest.

The remaining authors declare that the research was conducted in the absence of any commercial or financial relationships that could be construed as a potential conflict of interest.

The reviewer JL declared a shared affiliation with the author AM to the handling editor at the time of review.

## Publisher’s note

All claims expressed in this article are solely those of the authors and do not necessarily represent those of their affiliated organizations, or those of the publisher, the editors and the reviewers. Any product that may be evaluated in this article, or claim that may be made by its manufacturer, is not guaranteed or endorsed by the publisher.
