# Supplementary material for: Nanotechnology in combating biofilm: A smart and promising therapeutic strategy
Source: Front Microbiol. 2023 Mar 3;13:1028086. doi: 10.3389/fmicb.2022.1028086 (PMC10020670; doi:10.3389/fmicb.2022.1028086)
Supplement: Supplementary file 1 [file Data_Sheet_1.docx]

**Mechanisms of biofilm resistance to antimicrobial agents:**

Biofilm cells' sensitivity to antimicrobials is widely accepted. Higher osmolarity, oxygen limits, metal ion concentrations, and lower pH levels within a biofilm are responsible for gene expression and contribute shape of biofilm cell morphologies (Pitangui et al., 2012) The surface of a biofilm has more oxygen than the bottom and core (Hall-Stoodley et al., 2004). High-metabolic-activity cells are at the biofilm's surface, whereas slow-growing cells reside in the middle. Active cells recognize environmental changes and respond to antimicrobial stress. The majority of biofilm cells are metabolically inert and not developing. Stationary biofilm cells don't expand or respire and are more antimicrobial-tolerant (Antas et al., 2012). Changes in nutritional conditions and growth inhibition enhance biofilm drug resistance. Biofilms include intrinsic resistance mechanisms; but acquired and adaptive mechanisms also contribute to antibiotic resistance (Taylor et al., 2014). Biofilms help cells in adapting to their environment and increase antibiotic resistance. Multidrug resistance genes are expressed in response to alterations in the outer membrane proteins of cells within biofilms, which contributes to antibiotic resistance. For some antibiotics, the cells within biofilms can express resistance-related enzymes. In biofilms, alterations in the activity of multidrug efflux pumps contribute to bacterial resistance to antimicrobials. The greatest study has been done on the active efflux pumps of cells within biofilms. Dormant cells of the microbial subpopulation known as persister cells are phenotypic multidrug-tolerant variants rather than genetic ones and are therefore the predominant cell population in the stationary state of biofilm ecosystems (Al-Wrafy et al., 2022). The horizontal transfer of multidrug resistance genes is another mechanism by which cells within biofilms acquire antibiotic resistance and contribute to the evolution of those cells (Mah, 2012). QS plays a crucial role in mediating horizontal cell-cell transmission inside biofilms (Zhu et al., 2020). There is evidence to suggest that biofilms have evolved these processes as a general stress response that triggers the biofilm's resident microorganisms to respond to environmental changes (Mah, 2012). To fight biofilms, novel strategies targeting these mechanisms need to be developed. New approaches are needed to combat biofilms by targeting these systems.

**
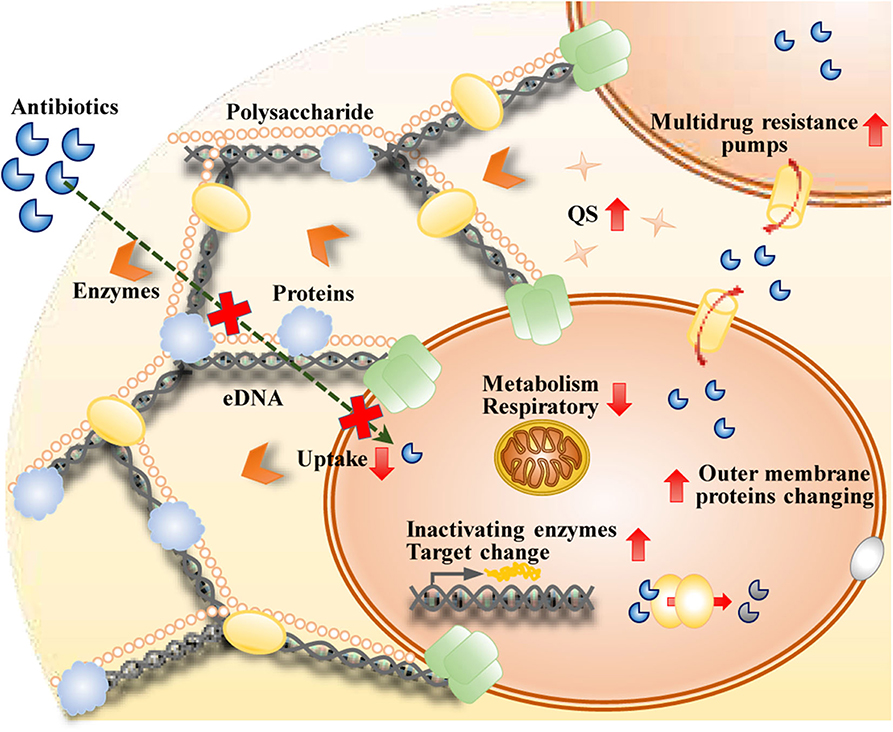
**

**Figure 1: Mechanisms of biofilm resistance to antimicrobial agents** (Zhang et al., 2020)**.**

**References**

Al-Wrafy, F. A., Al-Gheethi, A. A., Ponnusamy, S. K., Noman, E. A., and Fattah, S. A. (2022). Nanoparticles approach to eradicate bacterial biofilm-related infections: A critical review. *Chemosphere* 288, 132603. doi:10.1016/j.chemosphere.2021.132603.

Antas, P. R. Z., Brito, M. M. S., Peixoto, É., Ponte, C. G. G., and Borba, C. M. (2012). Neglected and emerging fungal infections: review of hyalohyphomycosis by Paecilomyces lilacinus focusing in disease burden, in vitro antifungal susceptibility and management. *Microbes Infect.* 14, 1–8. doi:10.1016/j.micinf.2011.08.004.

Hall-Stoodley, L., Costerton, J. W., and Stoodley, P. (2004). Bacterial biofilms: from the natural environment to infectious diseases. *Nat. Rev. Microbiol.* 2, 95–108. doi:10.1038/nrmicro821.

Mah, T. F. (2012). Biofilm-specific antibiotic resistance. *Futur. Microbiol.* 7, 1061–1072. doi:10.2217/fmb.12.76.

Pitangui, N. S., Sardi, J. C. O., Silva, J. F., Benaducci, T., Moraes da Silva, R. A., Rodríguez-Arellanes, G., et al. (2012). Adhesion of Histoplasma capsulatum to pneumocytes and biofilm formation on an abiotic surface. *Biofouling* 28, 711–718. doi:10.1080/08927014.2012.703659.

Taylor, E. N., Kummer, K. M., Dyondi, D., Webster, T. J., and Banerjee, R. (2014). Multi-scale strategy to eradicate Pseudomonas aeruginosa on surfaces using solid lipid nanoparticles loaded with free fatty acids. *Nanoscale* 6, 825–832. doi:10.1039/C3NR04270G.

Zhang, K., Li, X., Yu, C., and Wang, Y. (2020). Promising Therapeutic Strategies Against Microbial Biofilm Challenges. *Front. Cell. Infect. Microbiol.* 10. doi:10.3389/fcimb.2020.00359.

Zhu, L., Chen, T., Xu, L., Zhou, Z., Feng, W., Liu, Y., et al. (2020). Effect and mechanism of quorum sensing on horizontal transfer of multidrug plasmid RP4 in BAC biofilm. *Sci. Total Environ.* 698, 134236. doi:10.1016/j.scitotenv.2019.134236.
