# Supplementary material for: Nanotechnology in combating biofilm: A smart and promising therapeutic strategy
Source: Front Microbiol. 2023 Mar 3;13:1028086. doi: 10.3389/fmicb.2022.1028086 (PMC10020670; doi:10.3389/fmicb.2022.1028086)
Supplement: Supplementary file 2 [file Data_Sheet_2.docx]

**Table 1. Anti-biofilm properties of metal, metal oxide nanoparticles and nanocomposites.**

| **Nanoparticles** | **Synthesis Methods** | **Pathogens** | **Reference** |
| --- | --- | --- | --- |
| AgNPs | Synthesized by *Azadirachta indica* leaf extract | *Staphylococcus aureus* | (Namasivayam et al., 2013) |
| AgNPs | Leaf extracts of *Semecarpus anacardium*, *Glochidion lanceolarium*, and *Bridelia retusa* | *Pseudomonas aeruginosa, Escherichia coli, and Staphylococcus aureus* | (Mohanta et al., 2020) |
| AgNPs-PA | Purchased from ABC Nanotech Co. | PA01 planktonic bacteria and *Pseudomonas*  *aeruginosa* | (Park et al., 2013) |
| AgNPs-Ab | Reduction of silver nitrate | *Acinetobacter baumannii*, *P. aeruginosa*, *S. aureus*,  *Streptococcus mutans*, and *Candida albicans* | (Martinez-Gutierrez et al., 2013) |
| AgNPs-SP | Bioreduction of silver nitrate with *Allophylus*  *cobbe* leaves | *P. aeruginosa*, *Shigella flexneri*, *S. aureus*, and  *Streptococcus pneumoniae* | (Gurunathan et al., 2014) |
| AgNPs-Se | Reduction of silver nitrate with *Bacillus*  *licheniformis* biomass | *P. aeruginosa* and *Staphylococcus epidermidis* | (Kalishwaralal et al., 2010) |
| AgNPs-Ca | Reduction of silver nitrate with sodium citrate | *C. albicans* and *Candida glabrata* | (Monteiro et al., 2015) |
| AgNPs-Ti | Not mentioned | *S. epidermidis* | (Qin et al., 2014) |
| Ag-SiO2 | Stöber method | MRSA, *E. coli* | (Mosselhy et al., 2017) |
| AuNP-methylene | Turkevich–Frens method | *Candida albicans* | (Khan et al., 2012) |
| AuNP-NO | Reduction of HAuCl4  with trisodium citrate | *Pseudomonas aeruginosa* | (Duong et al., 2014) |
| Ultrasmall AuNPs | Not mentioned | *Staphylococcus aureus*, *Staphylococcusepidermidis*, *Escherichia coli*,and *P. aeruginosa* | (Boda et al., 2015) |
| AuNPs | Reduction of HAuCl4  with trisodium citrate | *S. aureus* and *P. aeruginosa* | (Sathyanarayanan et al., 2013) |
| ZnO | Wet chemical method | Classical (O395) and  ElTor (N16961) *V. cholerae* | (Sarwar et al., 2016) |
| ZnO | No information | *P. aerugonosa* | (García-Lara et al., 2015) |
| ZnO | Green synthesis | *E. coli,*  *P. aeruginosa* (ESBL), MRSA, MSSA | (Ali et al., 2016) |
| ZnO | Green synthesis | MRSA | (Vijayakumar et al., 2015) |
| ZnO | Purchased at the  Sigma–Aldrich | *M. smegmatis*,*M. bovis* BCG,  *E. coli, P. aeruginosa, S. aureus*,  MRSA | (Pati et al., 2014) |
| ZnO | Green synthesis | *V. cholerae*,  *E. coli* (ETEC) | (Salem et al., 2015) |
| ZnO | Purchased at the  Sigma–Aldrich | MRSA | (Jesline et al., 2015) |
| ZnO | No information | *E. coli* UTI89 andMG1655,  *K. pneumoniae* LM21,  MRSA SH1000,  *S. epidermidis* RP62A | (McGuffie et al., 2016) |
| ZnO | No information | *E. coli,*  *S. aureus,*  sobrinus ATCC 27352,  *Enterobacter* sp.,  *Marinobacter* sp. | (Wolska et al., 2015) |
| ZnO | Green synthesis | *C. violaceum* 12472,  *C. violaceum* CVO26,  *E. coli,*  *L. monocytogenes,*  *P. aeruginosa* PAO1 | (Al-Shabib et al., 2017) |
| ZnO | Microwave radiation | *C. violaceum* ATCC 12472,  *E. coli* ATCC 25922,  *P. aeruginosa* PAO1,  *K. pneumoniae* ATCC  700603, *S. marcescens*  ATCC 13880 | (Al-Shabib et al., 2018) |
| ZnO | Sol-gel method | R. dentocariosa,  R. mucilaginosa | (Khan et al., 2014) |
| ZnO | Purchased at US  Research  Nanomaterials Co | Uropathogenic *E. coli* | (Shakerimoghaddam et al., 2017) |
| Zn-doped  CuO | Sonochemical method | *E. coli* ATCC 25922,  *S. aureus* ATCC 29213,  *P. mirabilis* | (Shalom et al., 2017) |
| ZnO-NPs-So | Sol–gel | *Streptococcus oralis* | (Tabrez Khan et al., 2013) |
| ZnO-NPs-Rd | Sol–gel | *Rothia dentocariosa*  and *Rothia mucilaginosa* | (Khan et al., 2014) |
| ZnO-NPs-Pa | Sol–gel | *Pseudomonas aeruginosa* | (Sangani et al., 2015) |
| ZnO-NPs-Pasc | Soft chemical/  solution process | *Pseudomonas aeruginosa* | (Dwivedi et al., 2014) |
| ZnO-PvC-NPs | Nanophage | *Staphylococcus aureus* | (Seil and Webster, 2011) |
| ZnO-NPs-ec | Not mentioned | *P. aeruginosa*,  *Escherichia coli*,  and *S. aureus* | (Lee et al., 2014) |
| ZnOAu | Green synthesis | *S. aureus,*  *E. coli* | (He et al., 2014) |
| WO2 | Acid precipitation  routes | *B. subtilis* strain 168 | (Raie et al., 2017) |
| TiO2 | Green synthesis, by  bacteria | *B. subtilis* (FJ460362) | (Dhandapani et al., 2012) |
| TiO2 | Purchased at the  Sigma–Aldrich | MRSA | (Jesline et al., 2015) |
| TiO2-NPs-Ca | Hydrolysis of titanium  tetrachloride precursor | *Candida albicans* | (Haghighi et al., 2013) |
| TiNPs | Biosynthesis of titanium  nanoparticles using vaginal  *Lactobacillus crispatus* | *L. crispatus*, *Escherichia coli*, *Klebsiella*  *pneumoniae*, *Morganella morganii*, *Acinetobacter*  *baumannii*, and *Staphylococcus aureus* | (Ibrahem et al., 2014) |
| TiO-NPs as-syn | Acid-catalyzed | *Shewanella oneidensis* | (Maurer-Jones et al., 2013) |
| TiO-NPs P25 | Acid-catalyzed | *S. oneidensis* | (Maurer-Jones et al., 2013) |
| TiO2-NPs T-eco | Acid-catalyzed | *S. oneidensis* | (Maurer-Jones et al., 2013) |
| TiO2 | No information | MRSA bioﬁlm | (Jesline et al., 2015) |
| CeO2-CdO | Green synthesis | *P. aeruginosa* MTCC73 | (Maria Magdalane et al., 2017) |

**Reference:**

Al-Shabib, N. A., Husain, F. M., Ahmed, F., Khan, R. A., Ahmad, I., Alsharaeh, E., et al. (2017). Erratum: Biogenic synthesis of Zinc oxide nanostructures from Nigella sativa seed: Prospective role as food packaging material inhibiting broad-spectrum quorum sensing and biofilm. *Sci. Rep.* 7. doi:10.1038/srep42266.

Al-Shabib, N. A., Husain, F. M., Hassan, I., Khan, M. S., Ahmed, F., Qais, F. A., et al. (2018). Biofabrication of Zinc Oxide Nanoparticle from Ochradenus baccatus Leaves: Broad-Spectrum Antibiofilm Activity, Protein Binding Studies, and in Vivo Toxicity and Stress Studies. *J. Nanomater.* 2018. doi:10.1155/2018/8612158.

Ali, K., Dwivedi, S., Azam, A., Saquib, Q., Al-Said, M. S., Alkhedhairy, A. A., et al. (2016). Aloe vera extract functionalized zinc oxide nanoparticles as nanoantibiotics against multi-drug resistant clinical bacterial isolates. *J. Colloid Interface Sci.* 472. doi:10.1016/j.jcis.2016.03.021.

Boda, S. K., Broda, J., Schiefer, F., Weber-Heynemann, J., Hoss, M., Simon, U., et al. (2015). Cytotoxicity of Ultrasmall Gold Nanoparticles on Planktonic and Biofilm Encapsulated Gram-Positive Staphylococci. *Small* 11. doi:10.1002/smll.201403014.

Dhandapani, P., Maruthamuthu, S., and Rajagopal, G. (2012). Bio-mediated synthesis of TiO 2 nanoparticles and its photocatalytic effect on aquatic biofilm. *J. Photochem. Photobiol. B Biol.* 110. doi:10.1016/j.jphotobiol.2012.03.003.

Duong, H. T. T., Adnan, N. N. M., Barraud, N., Basuki, J. S., Kutty, S. K., Jung, K., et al. (2014). Functional gold nanoparticles for the storage and controlled release of nitric oxide: Applications in biofilm dispersal and intracellular delivery. *J. Mater. Chem. B* 2. doi:10.1039/c4tb00632a.

Dwivedi, S., Wahab, R., Khan, F., Mishra, Y. K., Musarrat, J., and Al-Khedhairy, A. A. (2014). Reactive oxygen species mediated bacterial biofilm inhibition via zinc oxide nanoparticles and their statistical determination. *PLoS One* 9. doi:10.1371/journal.pone.0111289.

García-Lara, B., Saucedo-Mora, M. A., Roldán-Sánchez, J. A., Pérez-Eretza, B., Ramasamy, M., Lee, J., et al. (2015). Inhibition of quorum-sensing-dependent virulence factors and biofilm formation of clinical and environmental Pseudomonas aeruginosa strains by ZnO nanoparticles. *Lett. Appl. Microbiol.* 61. doi:10.1111/lam.12456.

Gurunathan, S., Han, J. W., Kwon, D. N., and Kim, J. H. (2014). Enhanced antibacterial and anti-biofilm activities of silver nanoparticles against Gram-negative and Gram-positive bacteria. *Nanoscale Res. Lett.* 9. doi:10.1186/1556-276X-9-373.

Haghighi, F., Mohammadi, S. R., Mohammadi, P., Hosseinkhani, S., and Shidpour, R. (2013). Antifungal activity of TiO 2 nanoparticles against Candida albicans in vitro. *Infect. Epidemiol. Med.* 1.

He, W., Kim, H. K., Wamer, W. G., Melka, D., Callahan, J. H., and Yin, J. J. (2014). Photogenerated charge carriers and reactive oxygen species in ZnO/Au hybrid nanostructures with enhanced photocatalytic and antibacterial activity. *J. Am. Chem. Soc.* 136. doi:10.1021/ja410800y.

Ibrahem, K. H., Ibrahem, K. H., Salman, J. A. S., and Ali, F. A. (2014). EFFECT OF TITANIUM NANOPARTICLES BIOSYNTHESIS BY LACTOBACILLUS CRISPATUS ON UREASE,HEMOLYSIN&amp; BIOFILM FORMING BY SOME BACTERIA CAUSING RECURRENT UTI IN IRAQI WOMEN. *Eur. Sci. Journal, ESJ* 10.

Jesline, A., John, N. P., Narayanan, P. M., Vani, C., and Murugan, S. (2015). Antimicrobial activity of zinc and titanium dioxide nanoparticles against biofilm-producing methicillin-resistant Staphylococcus aureus. *Appl. Nanosci.* 5, 157–162. doi:10.1007/S13204-014-0301-X/TABLES/2.

Kalishwaralal, K., BarathManiKanth, S., Pandian, S. R. K., Deepak, V., and Gurunathan, S. (2010). Silver nanoparticles impede the biofilm formation by Pseudomonas aeruginosa and Staphylococcus epidermidis. *Colloids Surfaces B Biointerfaces* 79, 340–344. doi:10.1016/j.colsurfb.2010.04.014.

Khan, S., Alam, F., Azam, A., and Khan, A. U. (2012). Gold nanoparticles enhance methylene blue-induced photodynamic therapy: A novel therapeutic approach to inhibit Candida albicans biofilm. *Int. J. Nanomedicine* 7. doi:10.2147/IJN.S31219.

Khan, S. T., Ahamed, M., Musarrat, J., and Al-Khedhairy, A. A. (2014). Anti-biofilm and antibacterial activities of zinc oxide nanoparticles against the oral opportunistic pathogens Rothia dentocariosa and Rothia mucilaginosa. *Eur. J. Oral Sci.* 122, 397–403. doi:10.1111/eos.12152.

Lee, J. H., Kim, Y. G., Cho, M. H., and Lee, J. (2014). ZnO nanoparticles inhibit Pseudomonas aeruginosa biofilm formation and virulence factor production. *Microbiol. Res.* 169. doi:10.1016/j.micres.2014.05.005.

Maria Magdalane, C., Kaviyarasu, K., Judith Vijaya, J., Jayakumar, C., Maaza, M., and Jeyaraj, B. (2017). Photocatalytic degradation effect of malachite green and catalytic hydrogenation by UV–illuminated CeO2/CdO multilayered nanoplatelet arrays: Investigation of antifungal and antimicrobial activities. *J. Photochem. Photobiol. B Biol.* 169. doi:10.1016/j.jphotobiol.2017.03.008.

Martinez-Gutierrez, F., Boegli, L., Agostinho, A., Sánchez, E. M., Bach, H., Ruiz, F., et al. (2013). Anti-biofilm activity of silver nanoparticles against different microorganisms. *Biofouling* 29, 651–60. doi:10.1080/08927014.2013.794225.

Maurer-Jones, M. A., Gunsolus, I. L., Meyer, B. M., Christenson, C. J., and Haynes, C. L. (2013). Impact of TiO2 nanoparticles on growth, biofilm formation, and flavin secretion in Shewanella oneidensis. *Anal. Chem.* 85, 5810–5818. doi:10.1021/ac400486u.

McGuffie, M. J., Hong, J., Bahng, J. H., Glynos, E., Green, P. F., Kotov, N. A., et al. (2016). Zinc oxide nanoparticle suspensions and layer-by-layer coatings inhibit staphylococcal growth. *Nanomedicine Nanotechnology, Biol. Med.* 12. doi:10.1016/j.nano.2015.10.002.

Mohanta, Y., Biswas, K., Jena, S., Hashem, A., Abd_Allah, E., and Mohanta, T. (2020). Anti-biofilm and Antibacterial Activities of Silver Nanoparticles Synthesized by the Reducing Activity of Phytoconstituents Present in the Indian Medicinal Plants. *Front. Microbiol.* 11, 1–15. doi:10.3389/fmicb.2020.01143.

Monteiro, D. R., Takamiya, A. S., Feresin, L. P., Gorup, L. F., de Camargo, E. R., Delbem, A. C. B., et al. (2015). Susceptibility of Candida albicans and Candida glabrata biofilms to silver nanoparticles in intermediate and mature development phases. *J. Prosthodont. Res.* 59. doi:10.1016/j.jpor.2014.07.004.

Mosselhy, D. A., Granbohm, H., Hynönen, U., Ge, Y., Palva, A., Nordström, K., et al. (2017). Nanosilver–silica composite: Prolonged antibacterial effects and bacterial interaction mechanisms for wound dressings. *Nanomaterials* 7. doi:10.3390/nano7090261.

Namasivayam, K. R., S Christo, B. B., Karthigai Arasu, K Arun Muthu Kumar, S. M., Deepak, K., Karthick Raja Namasivayam, S., Karthigai Arasu, S. M., et al. (2013). Antibiofilm effect of biogenic silver nanoparticles coated medical devices against biofilm of clinical isolate of Staphylococcus aureus. *Glob. Journals Inc* 13.

Park, H. J., Park, S., Roh, J., Kim, S., Choi, K., Yi, J., et al. (2013). Biofilm-inactivating activity of silver nanoparticles: A comparison with silver ions. *J. Ind. Eng. Chem.* 19. doi:10.1016/j.jiec.2012.09.013.

Pati, R., Mehta, R. K., Mohanty, S., Padhi, A., Sengupta, M., Vaseeharan, B., et al. (2014). Topical application of zinc oxide nanoparticles reduces bacterial skin infection in mice and exhibits antibacterial activity by inducing oxidative stress response and cell membrane disintegration in macrophages. *Nanomedicine Nanotechnology, Biol. Med.* 10. doi:10.1016/j.nano.2014.02.012.

Qin, H., Cao, H., Zhao, Y., Zhu, C., Cheng, T., Wang, Q., et al. (2014). In vitro and in vivo anti-biofilm effects of silver nanoparticles immobilized on titanium. *Biomaterials* 35. doi:10.1016/j.biomaterials.2014.07.040.

Raie, D. S., Mhatre, E., Thiele, M., Labena, A., El-Ghannam, G., Farahat, L. A., et al. (2017). Application of quercetin and its bio-inspired nanoparticles as anti-adhesive agents against Bacillus subtilis attachment to surface. *Mater. Sci. Eng. C* 70. doi:10.1016/j.msec.2016.09.038.

Salem, W., Leitner, D. R., Zingl, F. G., Schratter, G., Prassl, R., Goessler, W., et al. (2015). Antibacterial activity of silver and zinc nanoparticles against Vibrio cholerae and enterotoxic Escherichia coli. *Int. J. Med. Microbiol.* 305, 85–95. doi:10.1016/j.ijmm.2014.11.005.

Sangani, M. H., Moghaddam, M. N., and Forghanifard, M. M. (2015). Inhibitory effect of zinc oxide nanoparticles on pseudomonas aeruginosa biofilm formation Inhibition of biofilm formation by zinc oxide nanoparticles. *Nanomed J* 2.

Sarwar, S., Chakraborti, S., Bera, S., Sheikh, I. A., Hoque, K. M., and Chakrabarti, P. (2016). The antimicrobial activity of ZnO nanoparticles against Vibrio cholerae: Variation in response depends on biotype. *Nanomedicine Nanotechnology, Biol. Med.* 12. doi:10.1016/j.nano.2016.02.006.

Sathyanarayanan, M. B., Balachandranath, R., Genji Srinivasulu, Y., Kannaiyan, S. K., and Subbiahdoss, G. (2013). The Effect of Gold and Iron-Oxide Nanoparticles on Biofilm-Forming Pathogens. *ISRN Microbiol.* 2013. doi:10.1155/2013/272086.

Seil, J. T., and Webster, T. J. (2011). Reduced Staphylococcus aureus proliferation and biofilm formation on zinc oxide nanoparticle PVC composite surfaces. *Acta Biomater.* 7. doi:10.1016/j.actbio.2011.03.018.

Shakerimoghaddam, A., Ghaemi, E. A., and Jamalli, A. (2017). Zinc oxide nanoparticle reduced biofilm formation and antigen 43 expressions in uropathogenic Escherichia coli. *Iran. J. Basic Med. Sci.* 20. doi:10.22038/ijbms.2017.8589.

Shalom, Y., Perelshtein, I., Perkas, N., Gedanken, A., and Banin, E. (2017). Catheters coated with Zn-doped CuO nanoparticles delay the onset of catheter-associated urinary tract infections. *Nano Res.* 10. doi:10.1007/s12274-016-1310-8.

Tabrez Khan, S., Ahamed, M., Al-Khedhairy, A., and Musarrat, J. (2013). Biocidal effect of copper and zinc oxide nanoparticles on human oral microbiome and biofilm formation. *Mater. Lett.* 97. doi:10.1016/j.matlet.2013.01.085.

Vijayakumar, S., Vinoj, G., Malaikozhundan, B., Shanthi, S., and Vaseeharan, B. (2015). Plectranthus amboinicus leaf extract mediated synthesis of zinc oxide nanoparticles and its control of methicillin resistant Staphylococcus aureus biofilm and blood sucking mosquito larvae. *Spectrochim. Acta - Part A Mol. Biomol. Spectrosc.* 137. doi:10.1016/j.saa.2014.08.064.

Wolska, K. I., Grudniak, A. M., Kamiski, K., and Markowska, K. (2015). “The Potential of Metal Nanoparticles for Inhibition of Bacterial Biofilms,” in *Nanotechnology in Diagnosis, Treatment and Prophylaxis of Infectious Diseases* doi:10.1016/B978-0-12-801317-5.00008-6.
